# Long-read genome sequence assembly provides insight into ongoing retroviral invasion of the koala germline

**DOI:** 10.1038/s41598-017-16171-1

**Published:** 2017-11-20

**Authors:** Matthew Hobbs, Andrew King, Ryan Salinas, Zhiliang Chen, Kyriakos Tsangaras, Alex D. Greenwood, Rebecca N. Johnson, Katherine Belov, Marc R. Wilkins, Peter Timms

**Affiliations:** 1 Australian Museum Research Institute, Australian Museum, 1 William Street Sydney, NSW, 2010 Australia; 2 Systems Biology Initiative, School of Biotechnology and Biomolecular Sciences, University of New South Wales, NSW, 2052 Australia; 3 Department of Wildlife Diseases, Leibniz Institute for Zoo and Wildlife Research, Berlin, Germany; 4 Department of Veterinary Medicine, Freie Universität Berlin, Berlin, Germany; 5 School of Life and Environmental Sciences, University of Sydney, Sydney, NSW 2006 Australia; 6 Ramaciotti Centre for Genomics, University of New South Wales, NSW, 2052 Australia; 7 Faculty of Science, Health, Education & Engineering, University of the Sunshine Coast, Locked Bag 4, Maroochydore DC, Qld, 4558 Australia; 8 Department of Translational Genetics, The Cyprus Institute of Neurology and Genetics, Nicosia, Cyprus

## Abstract

The koala retrovirus (KoRV) is implicated in several diseases affecting the koala *(Phascolarctos cinereus)*. KoRV provirus can be present in the genome of koalas as an endogenous retrovirus (present in all cells via germline integration) or as exogenous retrovirus responsible for somatic integrations of proviral KoRV (present in a limited number of cells). This ongoing invasion of the koala germline by KoRV provides a powerful opportunity to assess the viral strategies used by KoRV in an individual. Analysis of a high-quality genome sequence of a single koala revealed 133 KoRV integration sites. Most integrations contain full-length, endogenous provirus; KoRV-A subtype. The second most frequent integrations contain an endogenous recombinant element (recKoRV) in which most of the KoRV protein-coding region has been replaced with an ancient, endogenous retroelement. A third set of integrations, with very low sequence coverage, may represent somatic cell integrations of KoRV-A, KoRV-B and two recently designated additional subgroups, KoRV-D and KoRV-E. KoRV-D and KoRV-E are missing several genes required for viral processing, suggesting they have been transmitted as defective viruses. Our results represent the first comprehensive analyses of KoRV integration and variation in a single animal and provide further insights into the process of retroviral-host species interactions.

## Introduction

KoRV is a gammaretrovirus, most closely related to gibbon ape leukaemia virus (GALV)^1^ and is thought to be the result of an interspecies transmission^2^. It is considered to be a significant threat to the long-term survival of the koala. KoRV has been implicated in the pathogenesis of two major koala diseases, hematopoietic neoplasia^3–5^ and chlamydiosis^6^, the latter being endemic in the koala population. Several KoRV sub-types have been proposed, based primarily on the amino acid sequence of the receptor binding domain (RBD) of the viral envelope protein. There is strong evidential support for two common forms of KoRV: KoRV-A has been demonstrated in cellular expression and receptor interference assays to utilize the phosphate symporter (Pit-1) cell surface receptor during infection^7^, whereas KoRV-B utilizes the thiamin transport protein 1 (THTR1) receptor^5^. KoRV-A is presumed to represent the original cross-host transmitted strain^2^, is endogenized and is widespread in northern Australian koalas, which are thought to be 100% infected^8^. KoRV-B is a more recent retroviral subtype that is presumably the result of recombination, which, to date, has not been shown to be endogenized and is thought to be more virulent^5,6^. Many recently discovered additional variants (KoRV-C, D, E, F, G, H and I)^9–11^ have also been identified based on the RBD of the viral envelope protein, but as yet, no corresponding cell surface receptor has been identified^11^.

Whilst the variants of KoRV are becoming well-established, to date it has been difficult to examine the population of KoRV and KoRV-like insertions in any koala genome. Here we provide a detailed view of both the number and the diversity of KoRV integrants present in the genome of a single koala. We discover that KoRV-D and E have been integrated as incomplete provirus and suggest a mechanism that could explain their presence in this koala.

## Results

To assess all KoRV integration sites in a single koala, we analysed the whole genome sequence from a female koala, referred to as ‘Bilbo’, from a wild population. This individual was diagnosed with stage 3 chlamydial infection but at the time of euthanasia was free of detectable neoplasia’s. We recently assembled this genome (DDBJ/ENA/GenBank accession MSTS00000000) from 57x coverage PacBio sequencing of spleen tissue^12^. We used long-read sequencing technology for this assembly, due to its capacity to generate sequences of up to ~70 kb that carry full-length (8.4 kb) KoRV insertions and substantial flanking koala genome sequence. This provided a considerable advantage over Illumina short reads (e.g. 150 bp), which could not resolve the different KoRV insertion sites or types. We searched for KoRV in 1,906 contigs of the 3.19 Gb FALCON-assembled genome, in the 5,225 short alternate contigs that represent sequences that are heterozygous at any loci, and in all raw reads themselves. We found a total of 133 KoRV integration sites (Table 1; Supplementary Figure 1; Supplementary Table 1). Of these 133 integrations, 24 were in protein-coding genes. Disruption of any open reading frames seems unlikely because 22 of the integrations were in introns and two were in 3′ untranslated regions.

**Table 1:** KoRV integration sites found in the assembled koala genome or in unassembled PacBio long reads.

type	subgroup	completeness	in koala genome contigs	only in pacbio reads	total
KoRV	KoRV-A	full	48	19	67
		indel(s)	6	3	9
	KoRV-B	full	0	8	8
	KoRV-D	internal deletions	0	5	5
	KoRV-E	internal deletions	0	5	5
	cannot be determined	*env* region missing	5	1	6
recKoRV	recKoRV1	full	11	11	22
		indel(s)	1	3	4
	recKoRV2		1	0	1
	recKoRV3		2	0	2
solo LTR			2	2	4
total			76	57	133

### Characterization of integration site motifs

**Figure 1:**
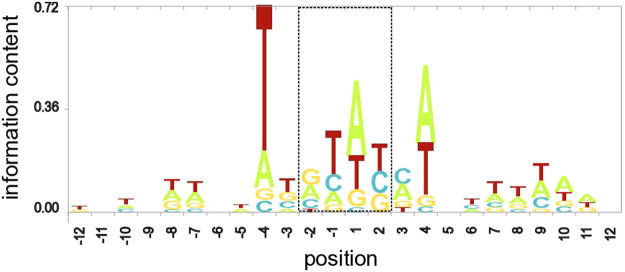
Sequence conservation at sites of KoRV and recKoRV integration into the koala genome. Conservation is graphically represented as a sequence logo with the height of each stack of letters corresponding to conservation at a position, and the height of each letter within a stack the frequency of that letter at the position (measured as information content; see ref.^30^). The logo was derived from a sequence alignment of 51 sites for which a 4 bp target sequence repeat was clearly identified (Supplementary Figure 1). The numbering is with respect to the centre of the 4 bp repeat (boxed).

In the koala genome assembly, for all but two cases, matching contigs were present that contained or did not contain the KoRV insertion at the same locus (Supplementary Figure 1). Alignment of these allelic sequences allowed us to define precisely each integration site (Supplementary Figure 1). This revealed a short (usually 4 bp but up to 6 bp) duplication of host DNA at the integration site, a feature that is typical of this class of retrovirus, and which has been previously observed for KoRV^2^. Alignment of the sequences at integration sites further revealed that the KoRV insertion sites are non-random, with a strong preference for T at positions −4 (Fig. 1). This appears to be part of a weak four base palindromic repeat (YBVH), consistent with reports of retroviruses integrating into palindromic DNA motifs^13^.

### Analysis of KoRV viral types

**Figure 2:**
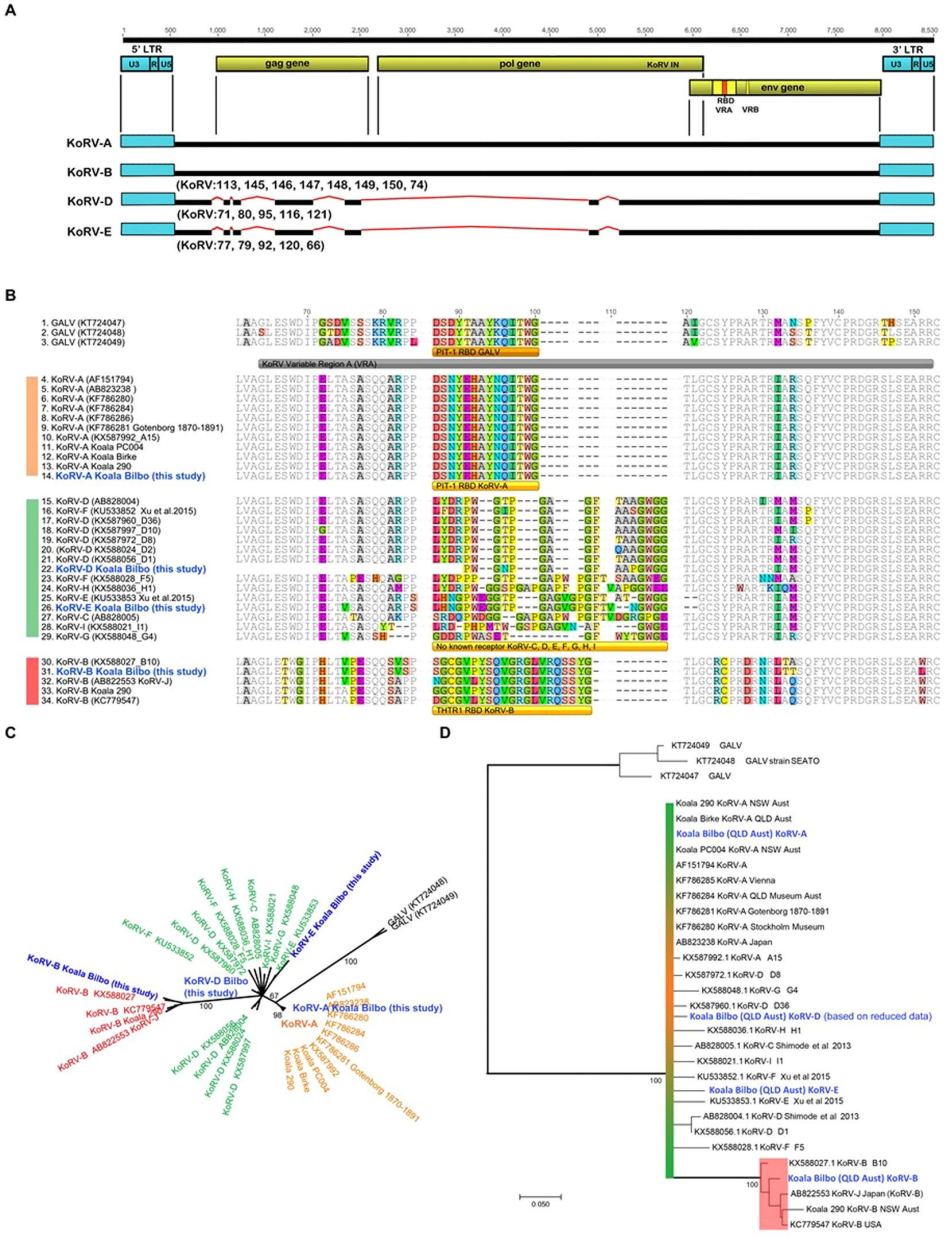
KoRV virus identified in the genome of the koala Bilbo, their structure, and relationship to other KoRV types. (**A**) KoRV-A and KoRV-B were both found in the genome as primarily full-length provirus. In contrast, all KoRV-D and KoRV-E provirus shared significant deletions of the gag and pol genes that would prevent processing and assembly of the virus. (**B**) Protein alignment of the envelope gene (env) of Gibbon Ape Leukemia Virus (GALV) and KoRV Variable Region A (VRA), showing 3 distinct and unrelated receptor binding domains that characterize the various KoRV subtypes. GALV and KoRV-A Receptor Binding Domains (RBDs) code for agonist of the Pit-1 cell surface receptor. The KoRV-B RBD codes for the agonist of the THTR1 receptor. The RBD for the group containing KoRV-C, D, E, F, G, H and I is characterized by a related but variable region with repeat motifs, deletions and substitutions. No cell surface receptor has been identified for any of these KoRV subtypes. (**C**) Analysis of the protein translation of the KoRV env VRA region from the genome of koala “Bilbo” in the context of previously published KoRV types. Galv, KoRV-A and KoRV-B form supported clades. GALV is most closely related to KoRV-A (they both use the PIT-1 receptor). Unfortunately, phylogenetic analysis of data containing unrelated receptor binding domains is unsuitable for resolving the evolutionary relationship of the published KoRV types. Analysis was performed in Genious Pro 5.4 using Treebuilder (Jukes Cantor model, NJ tree, 1000 Bootstraps, no defined outgroup). (**D**) Phylogenetic analysis of protein translation of the KoRV env VRA region with the receptor binding domain removed. This was done to assess the evolutionary relationship of the KoRV provirus types. This analysis indicates that KoRV-A, C, D, E, F, G, H, and I are closely related and that KoRV-B is a more recently evolved virus that is currently undergoing expansion. The evolutionary history was inferred by using the Maximum Likelihood method based on the Kimura 2-parameter model [1]. The tree with the highest log likelihood (−1731.9672) is shown. The percentage of trees in which the associated taxa clustered together is shown next to the branches. (Only branches with support > 60% are shown) Initial tree(s) for the heuristic search were obtained automatically by applying Neighbor-Join and BioNJ algorithms to a matrix of pairwise distances estimated using the Maximum Composite Likelihood (MCL) approach, and then selecting the topology with superior log likelihood value. A discrete Gamma distribution was used to model evolutionary rate differences among sites (5 categories (+G, parameter = 0.5017)). The tree is drawn to scale, with branch lengths measured in the number of substitutions per site. The analysis involved 31 nucleotide sequences. Codon positions included were 1st + 2nd + 3rd + Noncoding. There were a total of 377 positions in the final dataset, analyses were conducted in MEGA7 [2].

To assess the viral types present in this koala the genome assembly was used to look at the entire viral genomes for all KoRV subtypes present (Fig. 2A) and KoRV *env* gene specific PCR was been used to confirm some of these findings in low coverage examples. Variation at the nucleotide level was common for some KoRV subtypes and for this reason phylogenetic analysis has been performed on translated protein alignments. (Fig. 2B) The identification of KoRV virus sub-types was based on the receptor binding domain (RBD) of the KoRV envelope gene. (Pit1 in the case of KoRV-A or THTR1 in the case of KoRV-B). The RBD domain in the KoRV virus is part of the Variable Region A (VRA) of the envelope gene. Two methods were used to investigate the evolutionary relationship of the KoRV subtypes present in this koala (Fig. 2C,D).

### Assessment of endogenous vs exogenous integrations

**Figure 3:**
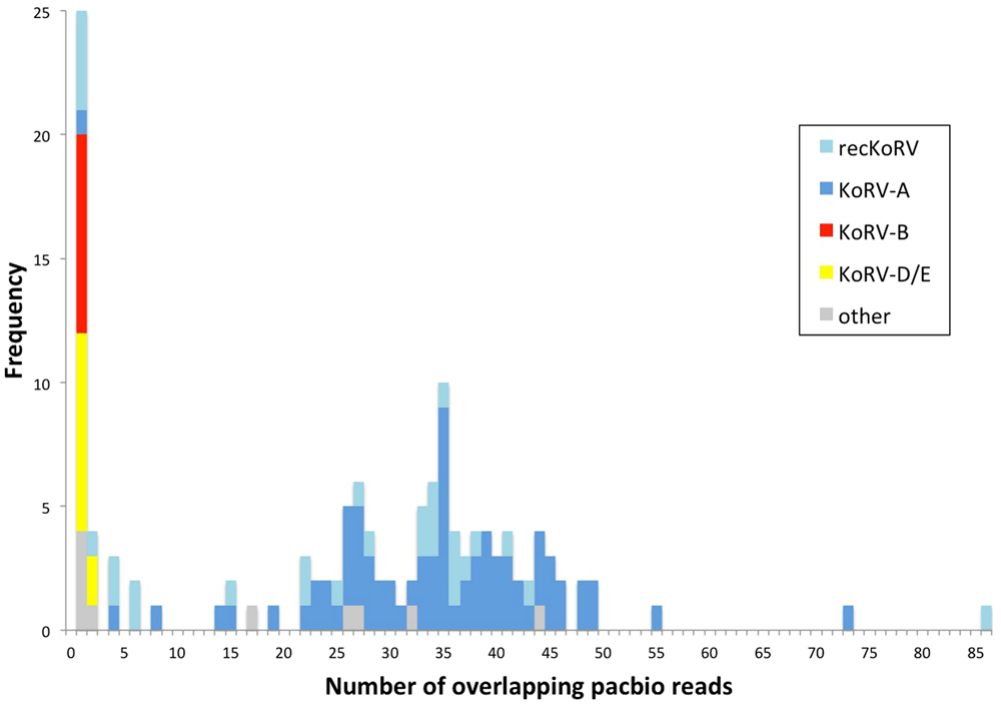
Depth of coverage of KoRV integration sites by PacBio reads. Sites with low coverage (1–2 reads only) are putative somatic insertions and those with 20× to 50× coverage are consistent with haploid coverage of germline insertions. The two sites with highest read coverage appear to be homozygous as they are the only sites whose pre-integration allelic sequence is not present in the Bilbo genome sequence assembly. KoRV type is shown in colour.

The genome sequencing was carried out to 57× coverage, which should result in a sequence depth of approximately 57 sequence reads for each and every part of the genome. When we analysed the depth of sequence coverage of all KoRV integration sites (Fig. 3), we observed a striking trend. While most KoRV integrations showed approximately 20× to 50× read depth (reflecting expected haploid or diploid nuclear genome coverage respectively), there was a second low coverage peak with a mode of 1× to 2×. The simplest explanation for this bimodality is that high read depth coverage is associated with germline integrations, while low read depth is the result of integration into somatic cells. Consistent with this, the median read depth (~35X) for the major peak is close to what we expect for haploid coverage (i.e. half the diploid assembly coverage value of 57X). It should be noted that the genomic DNA sequenced in this project was extracted from koala spleen but this organ is also known to act as a reservoir for monocyte cells. We cannot discern from our data if a particular cell source is responsible for the exogenous KoRV infections we observed.

### Endogenous integrations

We investigated the KoRV endogenous insertions of high read depth, focusing on 73 integrations which were within genome assembly scaffolds or contigs. These were briefly described as part of the koala genome sequence assembly analysis^12^ and fall into two groups: 58 insertions of KoRV-only sequences, and 15 insertions of a recombinant element, recKoRV (Fig. 3A)^14^.

### KoRV-A

**Figure 4:**
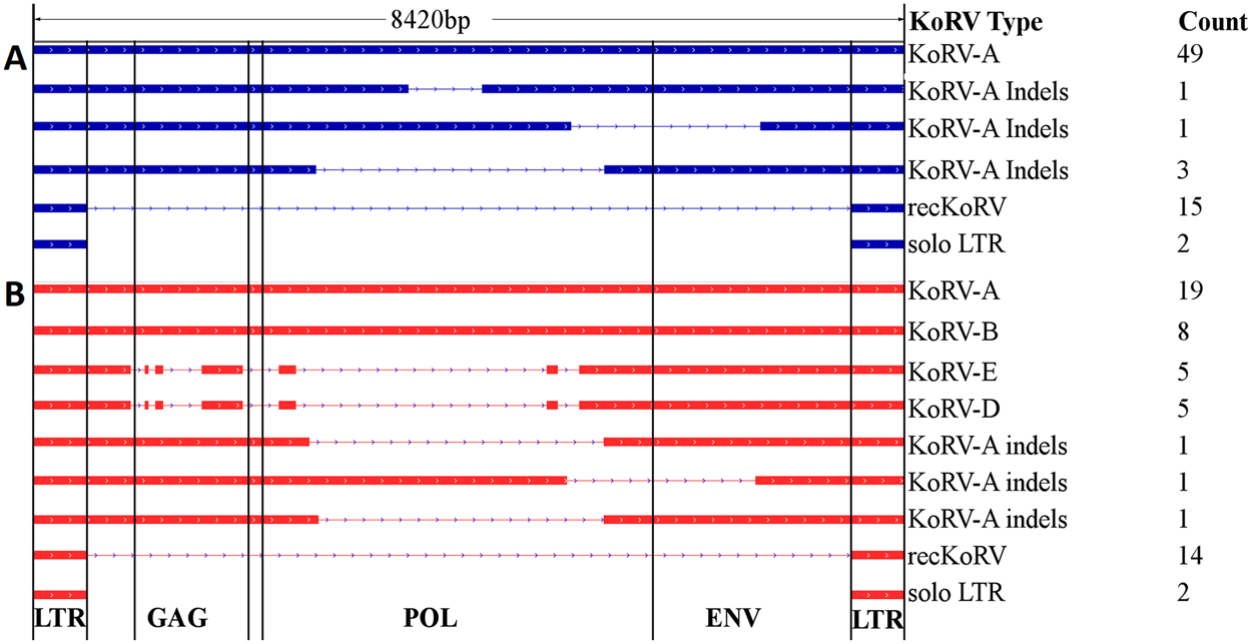
Structure of KoRV and recKoRV sequences. The *gag, pol* and *env* genes are shown as well as the LTRs that flank these genes. The non-KoRV component of recKoRV is not shown. (**A**) Forms found within the genome assembly, including both the primary and alternate contigs. (**B**) Forms found only within unassembled PacBio long sequence reads.

Of the 58 endogenous KoRV integrations present in the assembly, 48 were full-length KoRV-A sequences. These sequences showed > 99% sequence identity to the KoRV-A reference sequences (GenBank AF151794, AB721500). We were unable to confirm a complete set of intact open reading frames for *gag, pol* and *env* genes within a single provirus, but note that the frame shift insertions and deletions we observed were consistent with sequencing errors reported in single molecule sequencing technologies such as PacBio^15^. Due to higher error rate in long read technology, detection of low level variants within a population remains a limitation, even when combined with the short-read error removal (polishing) that was used in this genome assembly process^12^. Phylogenetic analysis of genome regions unaffected by read errors shows clear evidence of a recent expansion of KoRV-A in this individual when compared to the reference sequences (Supplementary Figure 2). The remaining endogenous integrations were seven KoRV fragments, and two solo long terminal repeat (LTR) sequences (Table 1; Fig. 4A).

### RecKoRV

**Figure 5:**
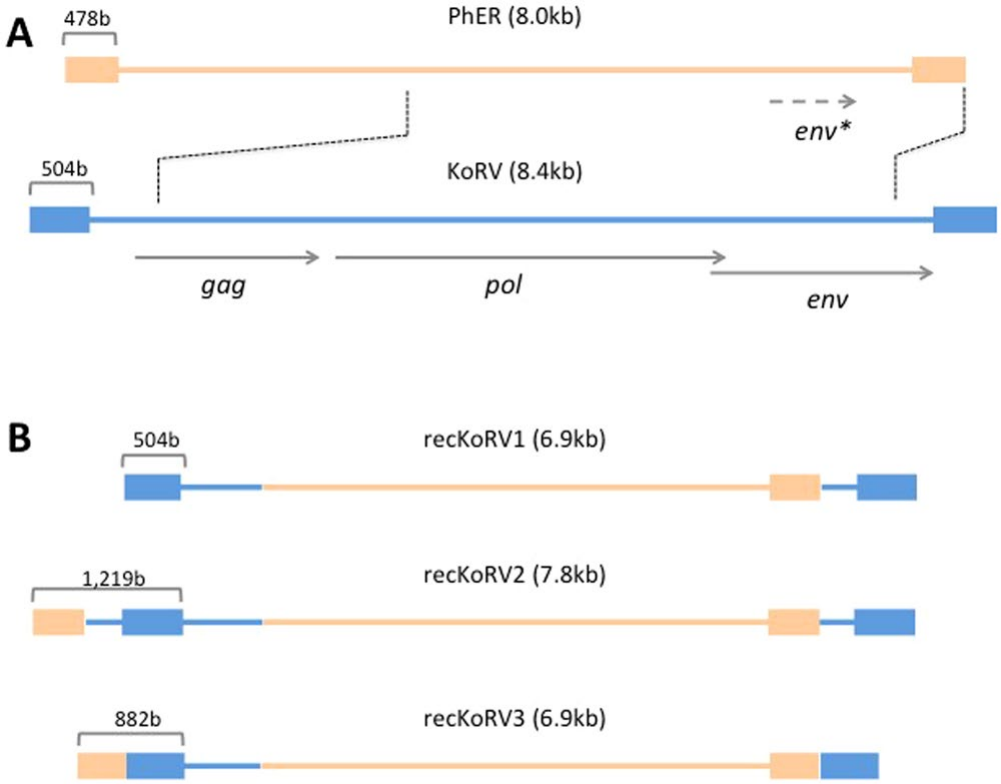
Recombination between KoRV and PhER can generate RecKoRV. (**A**) Parental structures. Dotted lines indicate putative break. PhER has no protein-coding capacity but has a region of low similarity to part of the KoRV env gene (env*) indicating a partially degraded gene (see Supplementary Figure 3). (**B**) Recombinant structures, which differ only in the composition of their terminal repeats.

A recombined KoRV (recKoRV) insertion was also observed 15 times in the genome of this single animal (Table 1). This element consists of left and right-hand segments of the KoRV genome, interspersed by a central non-KoRV sequence (Fig. 5A). Sequence similarity searches showed that this central sequence is part of a repeat structure (PhER; “Phascolarctos endogenous retroelement”^14^) that is abundant in the koala genome (results not shown). We characterised an exemplar of the repeat structure (Supplementary Figure 3). PhER is an 8 kb structure with some characteristics of an endogenous retrovirus (ERV) such as (a) direct terminal repeats of 478 bases, (b) a short sequence consistent with a role as a tRNA priming site for reverse transcription, and (c) limited sequence similarity to retroviral *env* gene sequences, although with no protein-coding capacity. Recombination between KoRV and PhER (Fig. 4A) appears to have given rise to a structure (recKoRV) comprising, (a) the KoRV 5′ LTR, *gag* leader sequence and truncated 5′ end of *gag* from KoRV, (b) the 4.9 kb 3′ end of PhER including its 3′ LTR, and (c) KoRV truncated 3′ end of *env* and 5′ LTR. We also noticed two less frequent variants of recKoRV (recKoRV2 and recKoRV3) whose terminal repeats have an arrangement that differs from that of the most frequent 6.9 kb form which we designate recKoRV1 (Fig. 5B). A question that arises from these observations is how the recKoRV element has been replicated and is present at 15 loci. Two possibilities are retro-transposition, and exogenous infection.

### Exogenous integrations

There were 57 further integration sites found in PacBio long sequence (raw) reads that were not assembled into the koala genome (Table 1; Fig. 4B). These were integration sites with low read depth (Supplementary Figure 1). 37 integration sites covered by only 1 or 2 reads are remarkable as they contain KoRV subtypes A, B, D and E. Of these 19 sites contain full length KoRV-A, 8 sites contained a full-length KoRV-B insertion, 5 sites contained KoRV-D and 5 sites contained KoRV-E integrations.

**Figure 6:**
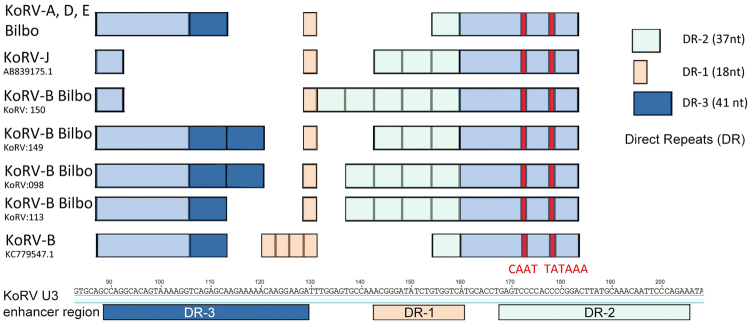
(**A**) comparison of the U3 LTR enhancer regions identified in koala “Bilbo” compared with published sequences. All KoRV-B U3 enhancer regions in koala “Bilbo” were novel LTR variants. The position and number of direct repeats DR-1 and DR-2 are indicated along with regulatory signals CAAT and TATAA box. DR-3 is a previously undescribed repeat region. The sequence and position of the direct repeats with respect to a KoRV-A reference sequence (AF151794). Minor sequence variation within these repeats was observed.

The origin of the somatic insertions of KoRV-A remain unresolved but an incomplete block of superinfection^2^ or retro transposition are two possibilities. The KoRV-B integrations were mostly full length, and were aligned to the reference sequence (GenBank KC779547). Analysis of the U3 enhancer region of KoRV-B LTR’s in koala “Bilbo” showed patterns of variation that have not been reported in previous studies of KoRV (Fig. 6). Two KoRV-B integrations show novel duplication of a 41 bp U3 region that we have termed direct repeat-3 (DR-3) and most show multimerization of the DR-2 motif^10^. Both these regions contain Glucocorticoid Receptor (GR) binding motifs. Glucocorticoids regulate numerous physiological responses including immunosuppression^16^. In all LTR regions we analysed only a single copy of DR-1 was observed. The limitations of low coverage PacBio sequences precludes high resolution analysis of these regions but multimerization of U3 enhancer elements would invariable lead to an increase in the number of transcription factor binding sites. Previous studies in other retroviruses, such as MuLV, FeLV and PERV, have reported similar repeat structures and have demonstrated that multimerization of LTR enhancer elements can increase viral replication^17^ and influence disease specificity and pathogenicity^18^. Gibbon ape leukemia virus (GaLV), porcine endogenous retrovirus (PERV), and murine and feline leukaemia viruses (MuLV, FeLV) are closely related to KoRV and these viruses are known to induce leukemia and immunodeficiency in their host species^19^.

Alignment of the KoRV-D and KoRV-E genomes to the KoRV-A genomic reference sequences (AF151794, AB721500) also revealed a surprising result. All KoRV-D and KoRV-E integrations contained identical deletions. Important viral elements necessary for independent replication were missing (Fig. 6A). The LTRs, Psi packaging region, integrase coding region and *env* gene, however, remained intact. The striking homology between the genomes of these KoRV types (D and E), found at multiple loci but absent as endogenous provirus strongly suggests a common progenitor and transmission as a defective virus. The presence of KoRV-A or KoRV-B integrations may provide helper virus functions, thereby enabling otherwise non-functional integrants to continue to replicate. Many gene therapy studies have shown that retroviruses will efficiently package viral RNA tagged with the appropriate Psi packaging signal^20,21^. It raises the interesting question of whether the KoRV subtypes (C, D, E, F, G, H, I)^14^ are all variations of defective KoRV. All these subtypes are characterized by an extremely variable receptor binding domain with amino acid duplications and deletions and as yet no study has been able to identify a corresponding cell surface receptor for these subtypes.

## Discussion

We have reported putative somatic integrations of five distinct forms of KoRV (KoRV-A, KoRV-B, KoRV-D, KoRV-E) as well as of recKoRV1. KoRV-A and recKoRV1 are also present in the germline, so somatic integration may have been caused by retro transposition, although recKoRV1 itself does not encode proteins required for reverse transcription or integration and so would not transpose autonomously. In the case of KoRV-A we cannot discount the possibility that an incomplete block of superinfection has led to exogenous infection. In contrast, we were unable to find germline (high copy number) versions of KoRV-B, KoRV-D or KoRV-E. KoRV-B has previously been described as an exogenous virus and our results support this in the individual koala analysed. By implication, KoRV-D and KoRV-E somatic integrations are also the result of exogenous infection, and since these two forms are themselves replication incompetent we suggest they have been transmitted as defective viruses.

Our comprehensive analysis of the integration of this retrovirus into its new host, the koala, gives us the opportunity to see first-hand, processes such as interspecies transmission, multimerization of repeat sequences in the LTR and recombination between different retroviruses, which have been reported for other retroviruses (FeLV, MuLV, PERV), but occurred millions of years ago, rather than in very recent times, as for the koala. Our genome sequence data provides direct evidence to support hypotheses that have been proposed for more ancient retroviral insertions, such as HERV^22^ and PERV^23,24^. At some time in the past, KoRV-A has co-opted the THTR1 cellular receptor binding site giving rise to the KoRV-B subtype and undergone expansion. This has led to variation not only in the envelope protein, but also the transcriptional regulatory regions in the LTRs. In addition, a pool of novel KoRV types has evolved, some, if not all of which, are defective. This diverse pool of viral variants in the same animal highlights the range of strategies being used by this retrovirus as it invades, or comes to equilibrium with its new host. From this study, it is clear that there still exists significant challenges to understanding the KoRV retroviral infection process. Poor limits of detection and difficulties discriminating KoRV types are two such challenges. In this work, we obtained our in-depth KoRV data from a single koala, which is a limitation of our study. However, obtaining sequence data from elements (such as retroviruses) that are repeated throughout the genome cannot be done with short read sequencing technology, which is why we used long read PacBio sequencing in our study (which currently has additional resource implications). It future, it will be essential to obtain similar data from an expanded range of koalas, from different geographical locations.

The fact that KoRV is rapidly evolving and diversifying has major implications for the long-term survival of the koala, particularly in relation to the other major infectious disease in this animal, *Chlamydia*. Recently, Waugh *et al*. (2017) showed that in a northern population of wild koalas (the same general region from which the animal in this study was obtained), infection with KoRV-B but not KoRV-A, contributed significantly to chlamydial disease pathogenesis. While it is accepted that chlamydial disease is generally more clinically severe in northern koala populations, chlamydial disease is also observed in southern koala populations. In a second 2017 study, Legione *et al*. (2017) analysed koalas from Victoria and found that while KoRV-B was not detectable in any of their animals, there was still evidence of chlamydial disease (though probably less severe). This confirms that while KoRV-B can be a contributing factor to more severe chlamydial disease, it is not the only risk factor. In fact, they reported that there was a clinically important increase in the odds of urogenital tract disease when *Chlamydia* plus KoRV were jointly detected. Both research groups did not observe an increase in *Chlamydia* infection in KoRV positive animals, but did see an increase in chlamydia disease, suggesting that the main factor might be the KoRV expression levels and any resultant immunosuppression. These observations suggest that as KoRV-A, B and potentially other more pathogenic KoRV types sweep through koala populations, we might expect to see worsening effects of chlamydial disease. This highlights the importance of understanding the complex mix of KoRV types present in an individual animal.

## Methods

### Koala whole genome sequencing and assembly

A full description of the whole genome sequencing of Bilbo DNA is provided in Johnson *et al*.^12^. Briefly, high molecular weight DNA from the spleen of koala Bilbo was sequenced on a Pacific Biosciences RS II platform (PacBio), using P6 C4 chemistry. A total of 272 Single Molecule Real-Time (SMRT) Cells were sequenced to give an estimated overall coverage of 57.3x based on a genome size of 3.5Gbp. DNA from Bilbo was also sequenced on an Illumina HiSeq X Ten platform, with 150 bp paired-end reads, yielding a minimum coverage of 34x. The overlapping layout consensus assembly algorithm, FALCON (v.0.3.0, https://github.com/PacificBiosciences/FALCON-integrate), was used to generate the genome assembly using PacBio reads. This resulted in an assembly into 1906 contigs representing homozygous regions of the genome. The genome polishing tool, Pilon^25^, was employed to error-correct the FALCON assembly with the Illumina reads and the genome was annotated according to Johnson *et al*.^12^. The koala genome is deposited at DDBJ/ENA/GenBank under the accession MSTS00000000. The version used in this paper is version MSTS01000000.

### Identification of KoRV sequences in whole genome sequence data

KoRV sequences within the scaffolds and alternative contigs of the phaCin_unsw v4.1 assembly of the Bilbo genome assembly were found using by using the program BLASTN^26^ to search with KoRV reference genome sequences (sequences (GenBank AF151794 and AB721500). To find KoRV sequences in the 171 GB dataset of Bilbo PacBio sequence reads we firstly searched using BLASR^27^ (version 1.3.1 with default parameters) to produce a set of 9,435 reads enriched in KoRV sequences. The enriched set was then searched using BLASTN^26^ using as queries KoRV-A and KoRV-B reference genome sequences (sequences (GenBank AF151794 and AB721500) as well as a recKoRV sequence from Löber *et al*.^14^. All BLASTN searches involving long read sequences, which have a high error rate, were done using a value of 6 for the word_size parameter. Search results were converted to BED format and the KoRV and recKoRV components of each read were merged with the program mergeBed, Reads not completely spanning an integration site were discarded. Pre-integration allelic sequences were found by using BLASTN^26^ to search the Bilbo genome sequence assembly with sequences flanking KoRV/recKoRV integrations as queries. In two cases the allelic site was not present in the Bilbo genome, but was found by searching the genome of another koala (Pacific Chocolate) described by Johnson *et al*.^12^. To check the expected relationship between pairs of allelic sequences we inspected dotplot alignments of representative sequences (not shown) created with the program dotter^28^. To define the small host sequence duplicated during integration, BLASTN^26^ was used to align the pre-integration site with 5′ and 3′ KoRV breakpoint sequences from the proviral sequence. For 51 sites with a clear 4 bp duplication (Supplementary Figure 1A), a ClustalW-formatted multiple sequence alignment was produced using muscle v3.8.31^29^. A logo, a graphical representation of sequence conservation at integration sites, was generated by skylign^30^ by uploading this multiple sequence alignment (in Supplementary Figure 1B) to the skylign webserver (http://skylign.org/). The skylign program used observed (unweighted) frequencies to estimate per-column parameters.

### Confirmation of KoRV subtypes using KoRV specific PCR

Primers UKoRV-F and UKoRV2-R (designed to conserved regions of the KoRV *env* gene) were used to amplify the predominant KoRV type present in the samples used in this study (in all positive samples tested this was KoRV-A).

The region amplified by these primers contains the surface GP70 subunit, VRA, VRB, CETTG motif, and the part of the transmembrane P15 region.

To screen Koala genomic DNA for KoRV-B type virus primers were designed to target regions that are specific to the KoRV-B receptor binding domains. These primers were matched to the UKoRV primers and produced overlapping contigs (total length ~ 470 nt). Primers were also designed against the RBD of KoRV-D and E insertions detected in raw PacBio reads from Koala Bilbo. Primers designed against previously published KoRV-D and E *env* gene failed to amplify so these primers were customised to amplify koala “Bilbo” KoRV-D and E specifically (~584 nt) (see Supplementary Figure 4).

PCR conditions were 1x MyTaq Red reagent buffer (Bioline Australia), 5 µM of each primer and 5U of MyTaq DNA polymerase (Bioline Australia). PCR cycling conditions were 94 °C for 3 min, 35 cycles of 94 °C for 20 s, 60 °C for 40 s and 72 °C for 40 s, and a final extension of 72 °C for 5 min. PCR product was checked using 1% TAE agarose gel electrophoresis and purified using ExoSap-IT (USB Corporation, USA). Purified PCR product was sequenced by the Australian Genome Research Facility (AGRF) using an AB-3730xl (Applied Biosystems, USA).

## Electronic supplementary material

Supplementary Table

Supplementary Figures
