# Supplementary material for: Long-read genome sequence assembly provides insight into ongoing retroviral invasion of the koala germline
Source: Sci Rep. 2017 Nov 20;7:15838. doi: 10.1038/s41598-017-16171-1 (PMC5696478; doi:10.1038/s41598-017-16171-1)
Supplement: Supplementary file 2 — Supplementary Figures [file 41598_2017_16171_MOESM2_ESM.pdf]

SUPPLEMENTARY DATA

Long-read genome sequence assembly provides insight into ongoing retroviral invasion of the koala germline

Matthew Hobbs, Andrew King, Ryan Salinas, Zhiliang Chen, Kyriakos Tsangaras, Alex D. Greenwood, Rebecca N. Johnson, Katherine Belov, Marc R. Wilkins and Peter Timms

Supplementary Figure 1

Characterisation of 51 integration sites at which a 4bp duplication is clearly observed.

A. Alignments pre-integration and proviral allelic sequences. The program blastn was used to align the pre-integration site (the query sequence) with 5’ and 3’ KoRV breakpoint sequences from the proviral sequence (the subject sequence).

```
Query_1: phaCin_unsw_v4.1.fa.scaf00011       Subject_1: 000010F-150-01     KoRV:10


Query_1                                                                                        4328289
TTAAATAGAGAAGAAACAAATTTCTGTTTTGGTCAAAACTTTGAAGGGGTGctatCACTACAATTTCAATGTGTCAGGTACAATACATTCTAGG
AGTTCAGCACT  4328393

Subject_1       1043                .......................................................
1097

Subject_1
9529                                                                ..................................
....................  9582


Query_1: phaCin_unsw_v4.1.fa.scaf00232       Subject_1: 000250F-036-01     KoRV:40


Query_1                                                                                        4117747
GTCCTATAGTTAGTATGTGTCAGAAGCAGGACTTGAACCCAGGTCTTCCTGactcCAGAACCAGTTCTCTACTCTGCCACCCTGCTGCTATTGC
TCAAGATCAGT  4117851
```

Subject_1 810 ....
864

Subject_1
9296 ....
.... 9349

Query_1: phaCin_unsw_v4.1.fa.scaf00011 Subject_1: 000010F-003-01 KoRV:9

Query_1 16123887
TGCGCAATTATAGCAGTAATCAGATCAAAAAGAATGGCTGGAGCTACATTgcgaAAAGCTCCAAATGGTGAGCACAGGAGTTTATGTGTAGCAT
GTAGTGCCCC 16123991

Subject_1 26476 ....
26530

Subject_1
34964 ....C.................
.... 35017

Query_1: phaCin_unsw_v4.1.fa.scaf00021 Subject_1: 000018F-236-01 KoRV:12

Query_1 7206236
GGGAAGGAGAGGGAGAGGGAGGGGGAGAAAAATCTAAAACTCAAAAGCTTatagAAGTGATTGCTAATAATTCAAAATAAATTAATTTTTAAAA
A-GGGTAGCCA 7206339

Subject_1 1261 ....
1314

Subject_1
9745 ....
...........A......... 9799

Query_1: phaCin_unsw_v4.1.fa.scaf00019 Subject_1: 000020F-232-01 KoRV:13

Query_1 2787230
GAACTTTTTGGAGGTTCACCCATCACTAATTTTCAGGTCCACTGGAGCATTacatGACTTGCTTTCAACCTTTTCTACAAATTCAGAACCTTTT
CTACAAATTCA 2787334

Subject_1 21671 ....
21725

Subject_1
30162 ....
.... 30215

Query_1: phaCin_unsw_v4.1.fa.scaf00048 Subject_1: 000048F-034-01 KoRV:19

Query_1 1749198
GAAAAGCCTAGCTAAATTGAGAGAGGTTTATCAACAGGTGAGTTGCAAGGTaaatGATTTTTGAGCCAAAGAATCTCTCGAAGGCTATCTTCTA
AGTCTTAGAGA 1749302

Subject_1 1162 ....
1216

Subject_1
9653 ....
.... 9706

Query_1: phaCin_unsw_v4.1.fa.scaf00001 Subject_1: 000000F-381-01 KoRV:2

Query_1 21821686
GAATCCAAGCAGAATTCCTGAAGTGCCTCCCTATCTATATTTAACTTTCAAgggtCAAAAATTAAGGGAAACCAACTTAAAATGTCTATCCTTC
TCAACTGTTAT 21821790

Subject_1 1290 ....
1344

Subject_1
9779 ....
.... 9832

Query_1: phaCin_unsw_v4.1.fa.scaf00048 Subject_1: 000048F-177-01 KoRV:21

Query_1 4198036
GACCACTAAAAAAAAAAATAAAAGAACACAAATGAATTTCATTGTTTTGTAGgttcCTTCTTTGTAGAAACCCAACAGATTTATGAACATTAGAA
AAATACTATTT 4198140

Subject_1 8485 ....
8539

Subject_1
16968 ....
.... 17021

Query_1: phaCin_unsw_v4.1.fa.scaf00100 Subject_1: 000104F-043-01 KoRV:28

Query_1 3756663
AGTGAAATGAGCAGGACCAGGAGAATGTTGTACACAGTAACAGCCATGTTGtgcgATGACTAATGTTGATAAACTTAACCCTTCTCAGCAATAC
AAGGATCTAAG 3756767

Subject_1 2697 ....
2751

Subject_1
11186 ....
.... 11239

Query_1: phaCin_unsw_v4.1.fa.scaf00119 Subject_1: 000127F-087-01 KoRV:30

Query_1 2664670
CCACCATCCATAATCCTTCCTATCCTAGACCCTTCCTAACTTTCCAGTCTGcttaCACCTTATTCACCTTCATGTACTCGTCAATCCAGTGACA
CCAGCTGACTT 2664774

Subject_1 1092 ....
1146

Subject_1
9580 ....
.... 9633

Query_1: phaCin_unsw_v4.1.fa.scaf00228 Subject_1: 000245F-025-01 KoRV:39

Query_1 1170867
TAGATTGTTGGAGTTGGTGTCTGTGAACATTCTAAAGTAATGATTATATTGgtagATCATGCTAGTTACTTCAAAACAAAGCTATATAACTCAT
ATAACTAGGAT 1170971

Subject_1 1274 ....C............................
1328

Subject_1
9761 ....
.... 9814

Query_1: phaCin_unsw_v4.1.fa.scaf00323 Subject_1: 000351F-010-01 KoRV:46

Query_1 1318950
GGTTCCTCTACATGTTAGCTTCTTCCCATTTTTTTTTCTCCCTTCAAGGTCctttCTGTGAAGAGAATCTAGAAGATGGAAGCCAGTAGACTAG
AATCTCTCTTC 1319054

Subject_1 19071 ....
19125

Subject_1
27555 ....
.... 27608

Query_1: phaCin_unsw_v4.1.fa.scaf00003 Subject_1: 000002F-035-01 KoRV:5

Query_1 8272310
TTTGTGAGCTTCCACTAGTAGTTCCTCTTCTCTAAAGTCTCTTGCCCCATTattgTATGGCTGAGATTTCCCTGCCAAGCCTCAGCCTTGGATC
ACTCACACAGT 8272414

Subject_1 2872 ....
2926

Subject_1
11360 ....
.... 11413

Query_1: phaCin_unsw_v4.1.fa.scaf00034 Subject_1: 000033F-003-01 KoRV:16

Query_1 6069669
CTTTACATAGGCTGTGCCCTCTTCCAATGTATTCCTCCTTATCTCTCACTCatagATTCCATAACTTTGTACAAAGCTCAACTCAAGAGCATCA
TCCTTCATTTG 6069773

Subject_1 27756 ....
27810

Subject_1
35526 ....
.... 35579

Query_1: phaCin_unsw_v4.1.fa.scaf00085 Subject_1: 000088F-067-01 KoRV:24

```
Query_1                                                                                        7732177
TGCCGGGCAGGACTCTAACCAAGAGAAGCACGACTTTGTTCAAAAGTAATGctacAGGCATAACTGGCAAATATTTTTGAAACTTTCTGAGCCA
CACTTTCAGAA  7732281

Subject_1       2440                  .......................................................
2494

Subject_1
10925                                                       ..................................
....................  10978


Query_1: phaCin_unsw_v4.1.fa.scaf00246       Subject_1: 000264F-004-01     KoRV:43


Query_1                                                                                         828254
AAAATGTGGTACCTGGAGATGAACACAGAAATGATCTGACTAGGCCCCTGTgtacATGCTGAGAGCATCGCTTCCCTCATTCTGGACATTATAC
ATGTATTAATA  828358

Subject_1      573                    .......................................................
627

Subject_1
9059                                                        ..................................
....................  9112


Query_1: phaCin_unsw_v4.1.fa.scaf00380       Subject_1: 000406F-009-01     KoRV:48


Query_1                                                                                          20667
CATAAGGAGTAATAGAGAAAATGAGGACAATAAAGGTAAAAAGCCAGGTAGgtagGTTGAGTAAGAAACCAAAGCAACTGAACAAAAAAAAGGA
GGGGAGACGGT  20771

Subject_1       1575                  .......................................................
1629

Subject_1
7925                                                        ..................................
....................  7978


Query_1: phaCin_unsw_v4.1.fa.scaf00003       Subject_1: 000002F-358-01     KoRV:6


Query_1                                                                                       10153609
TAGAATATAATCTGTGTTATGAGGTCATAGATTAAACACTGGAAGGGTTCttagAAGCTATAGAGTCCAATATTCTCGTGATAAAAATATGAAA
ACATCTAGA  10153712
```

Subject_1   39   ....
93

Subject_1
8514   ....
.... 8566

Query_1: phaCin_unsw_v4.1.fa.scaf00009   Subject_1: 000008F-100-01   KoRV:7

Query_1   3604958
AAGGACGAAGTGACAAGGCCCTATAATCTCCAGTTTAGAGTGCATGTCACTgcctCAACCTTCTTCTCCTGTGCCCCATTCCAACTCTGCCCAC
TCATTCTACTG 3605062

Subject_1   146724   ....
146778

Subject_1
155207   ....
.... 155260

Query_1: phaCin_unsw_v4.1.fa.scaf00009   Subject_1: 000008F-127-01   KoRV:8

Query_1   8429833
CAGCTATTTGGCCTAATCCAAATTACTGCCTCCATTCTGCACTTGGCTTTGgcagTAGGCTTAGAAAATTTGGTTCTTTCACTCTTCTAGCTGA
AAAAAGAAAAA 8429937

Subject_1   3657   ....
3711

Subject_1
9355   ....
.... 9408

Query_1: phaCin_unsw_v4.1.fa.scaf00013   Subject_1: 000012F-250-01   KoRV:11

Query_1   20996513
ACTTTTCTAAGTCTCAGTTTTCTCATCTAAAAAAAATGGGGATAACATTatacTATTTACCTCACAGGACTGTGAGTCAAATACTATGTAAATG
CAAGGTTCT 20996615

Subject_1   1149   ....-........................
1200

Subject_1
9632 ....
.... 9685

Query_1: phaCin_unsw_v4.1.fa.scaf00022 Subject_1: 000021F-100-01 KoRV:14

Query_1 3649515
AAATTTGGTCCTTTGTGGAGGAAAGTGAGTGGGAATTTCAGGTCAGAAAACcagcAAAGTTTCCATTCCTCTTCAGTTTTTTTTTTAAGACTTT
CACAAATAAAT 3649619

Subject_1 489 ....-..
542

Subject_1
8971 ....
.... 9024

Query_1: phaCin_unsw_v4.1.fa.scaf00027 Subject_1: 000027F-033-01 KoRV:15

Query_1 9770555
GTACCACTTAGTTATCCTACCTGGCACACAGTAGGCACTTAAATGTTTATTgaacTAAGGGAAGGAAGAAGTATTTATTGTCTACTACATGCCA
GGCACT 9770654

Subject_1 5350 ....
5404

Subject_1
13830 ....-..........................
.... 13877

Query_1: phaCin_unsw_v4.1.fa.scaf00048 Subject_1: 000048F-115-01 KoRV:20

Query_1 8189611
ATATACATACACACAAATATACTTACAAATATATGTATGGATATATATTGTgtatCTATATATTTATGTGTACATACACACACATACATATCCA
AAGATACATGT 8189715

Subject_1 4248 ....
4302

Subject_1
4805 ....
.... 4858

Query_1: phaCin_unsw_v4.1.fa.scaf00058 Subject_1: 000062F-078-01 KoRV:22

Query_1 9863877 GAAATTTTTTGAGTCCTACCATTTTT-
AAAAGCAACCCATAGTGGTTTGTTgatgGATTCTTGTTTCCTGTGAAAAGGTGCTTCTAAAAGCCATTTTTTTCTTCT 9863981

Subject_1 4174 ....A.............................
4229

Subject_1
12708 ....
.... 12761

Query_1: phaCin_unsw_v4.1.fa.scaf00087 Subject_1: 000090F-011-01 KoRV:25

Query_1 7718611
TCTTTTTCATTTCATGGTTGGGAAATCAGGCTACCTGTTATGTTTAATATGctacCTATATTATAAATATTACTTACATGTATAGATATGTGTA
GTACGCATATG 7718715

Subject_1 2895 ....
2949

Subject_1
11369 ....
.... 11422

Query_1: phaCin_unsw_v4.1.fa.scaf00091 Subject_1: 000094F-117-01 KoRV:26

Query_1 4490914
AAACCCTTAGAGCCCTCAATTATCAATCCACTATCGAGAAGCATTTATTAAgtgcCTCTTATATGACAGGACTATGTAGGCACCATGTCTTTAT
TCCTGCTTGCA 4491018

Subject_1
27114 ....
.... 27167

Subject_1 26570 .............................-......................-......
26622

Query_1: phaCin_unsw_v4.1.fa.scaf00092 Subject_1: 000095F-062-01 KoRV:27

Query_1 7537568
CAAGTCAAGACATCCCCATGATGTCATTGGTCCTCTTCAAACATTAAGGACaaacAACAATAATGACAACAACCAGGGACCCAGCCTGACTTAG
GCCCACACTAT 7537672

Subject_1 6214 ....
6268

Subject_1
14730 ....
.... 14783

Query_1: phaCin_unsw_v4.1.fa.scaf00001 Subject_1: 000000F-386-01 KoRV:3

Query_1 28268207
TTGTATTTAAGATAGGCTGGAATTCAAATCCAACCTCAGACATGTTAGTTgtatGACCCAGGGCAAATCACTTAACCTCTGTCTTCCTCAGCTC
TCTCATCTAT 28268311

Subject_1 2457 ....
2511

Subject_1
10938 ....
.... 10991

Query_1: phaCin_unsw_v4.1.fa.scaf00130 Subject_1: 000137F-092-01 KoRV:31

Query_1 6393442
GCATATCCAGTCTTGTTTTCAGATCCCTTTACAGTCTTATTTCAAAAAATCtttcATCCAACAGCACTAGACACAGACCTGCCCCTGACCATGC
TACAGCATCTC 6393546

Subject_1 5534 ....
5588

Subject_1
14012 ....
.... 14065

Query_1: phaCin_unsw_v4.1.fa.scaf00138 Subject_1: 000147F-028-01 KoRV:33

Query_1 2629750
ATTGTGCTAAAAGAAATGATAAAAGAGGAGGTTTCAGTGAGGCCTGGGAAGtctcCTATGACCTGATGGAAAGTGAAGGGAGCAGAACAAGGAG
AATTATTTATA 2629854

Subject_1 1006 ....
1060

Subject_1
9488 ....
.... 9541

Query_1: phaCin_unsw_v4.1.fa.scaf00150 Subject_1: 000159F-025-01 KoRV:34

Query_1 3463542
ATATTAAATAACATATTAGCAAAGAGATTACAGTAACATATCACAAATATCatacAATATGACCAGGTTGGATTTATAACAGAAATAGAGGGTT
GCTTCAATATT 3463646

Subject_1 982 ....
1036

Subject_1
9467 ....
.... 9520

Query_1: phaCin_unsw_v4.1.fa.scaf00150 Subject_1: 000159F-031-01 KoRV:35

Query_1 3620412
GCTTCTGCTACTCATCAGCTGTGTGACCTTGGTAAGTCATTTCAGTTCTTTggatATCAGGTTCCTCATTTATTGAATGAAGTGGATGGACTCA
ATGGTCTTGAA 3620516

Subject_1 1040 ....
1094

Subject_1
4815 ....
.... 4868

Query_1: phaCin_unsw_v4.1.fa.scaf00239 Subject_1: 000257F-028-01 KoRV:42

Query_1 1147085
TTTTTGATGATGGCCACAGAATAATGGGGGGGAAAAGCAAGGGAAGATTTTacagCATCTACAAACATTAATTTAACTGTTAATACTCCATATT
TGAGATCTTTA 1147189

Subject_1 11591 ....A.......................
11645

Subject_1
18710 ....
.... 18763

Query_1: phaCin_unsw_v4.1.fa.scaf00357 Subject_1: 000387F-001-01 KoRV:47

Query_1 144912
GCATTTAGACATTGATATCTTCCTGTGCTAAAGGTTCCGTGCAGTTTGGTTacagATGCTTAGATTAAATGCTACCTAATATTCATTGGATCTT
AGTGTCCAGAA 145016

Subject_1 14134 ....A..............................
14188

Subject_1
22618 ....
.... 22671

Query_1: 000022F-201-01 Subject_1: phaCin_unsw_v4.1.fa.scaf00023 KoRV:51

Query_1 1434
GACCTCACCCAACCTGAGAAGAGAAAGCTGTTCAAATATGCTCCAGTCTTGaaatGAGCTAAGAGAGCTTAGTCTAAGGGCAGAGAATGGATGA
CGAGTATCATT 1538

Subject_1 284602 ....
284656

Subject_1
293089 ....
.... 293142

Query_1: 000027F-115-01 Subject_1: phaCin_unsw_v4.1.fa.scaf00027 KoRV:52

Query_1 21304
TGTGATTATTTCAGCCCTTCCACAGCTCCTCATGCTGTGAAATCTTTGGCCatggGTTTCTATAAATCCTCCCTCCA--AA-
GGTCTGCTCAGAGTTATTTGAATCAG 21408

Subject_1 2807222 ....
2807276

Subject_1
2815708 ..........................TA..G...
.... 2815764

Query_1: 000051F-059-01 Subject_1: phaCin_unsw_v4.1.fa.scaf00050 KoRV:53

Query_1 34872
ATGTTAAAAGACCTGGAGGGAGGAGGTGAGTGACTCCTCTATGGAGACTTTgtacAGTGGCAAGGGTGGTGAGGACCACATAGGAGAAAGAAGG
AGAAAGCACGG 34976

Subject_1 4443888 ....
4443942

Subject_1
4449510 ....
.... 4449563

Query_1: 000114F-002-01 Subject_1: phaCin_unsw_v4.1.fa.scaf00108 KoRV:56

Query_1 5629
ACACTCCCATCCAAGATCTTAGGGGACACATAAAACTGCAAGAGTTCCTTCatgtCTGCTGAAGTGGGATCAGCGATTTTATAGTGCTCTTGGG
GGATGGGGAGA 5733

Subject_1 8565408 ....
8565462

Subject_1
8572009 ....
.... 8572062

Query_1: 000172F-016-01 Subject_1: phaCin_unsw_v4.1.fa.scaf00164 KoRV:57

Query_1 2356
GTTTGGGTTCCATGTTTCTTTTTTTTGAGTAGATGCTATGGATTTATTTTTccttGTCTTATAATTTGGTAGACTGTGACATAGAAGAGAACAG
CAAAGTCAAAA 2460

Subject_1 5996344 ....
5996398

Subject_1
6004826 ....
.... 6004879

Query_1: 000311F-041-01   Subject_1: phaCin_unsw_v4.1.fa.scaf00288   recKoRV:10

Query_1 19456
GAAGAACCTAGGTTAAGAACATATAACAAATCTGTGGCAATCCTGGCAAAGggatCGTGTAGTCTGCAATAATAATAATTGTAGTTGATGTTTA
GTTCTGTTTAG 19560

Subject_1   726445   ....
726499

Subject_1
733331   .......A....G..................T..
.... 733384

Query_1: 000387F-001-01   Subject_1: phaCin_unsw_v4.1.fa.scaf00357   recKoRV:11

Query_1 4043
TTTCAGTTGGTTCTGAAATTGTAGCAGTTGAGCTAAGCCATTGGAGTTCTTgtttCCAAAGCACATTTGTTGTGTAGCTGAGGGATGAAGCAGG
GATTGCTAAGG 4147

Subject_1   127967   ....
128021

Subject_1
134853   ....
.... 134906

Query_1: phaCin_unsw_v4.1.fa.scaf00069   Subject_1: 000071F-005-01   recKoRV:2

Query_1 1146096
CTCATTGGCTTTCCCCAGCCTCAGTTTCCTCATCTGCAAAATAAGAGCATCagatCAGATGATCTCTAAGTCCCTTCCAACCTGAGCAACACAT
GGATCTATAGA 1146200

Subject_1   23451   ....
23505

Subject_1
30350   ....
.... 30403

Query_1: phaCin_unsw_v4.1.fa.scaf00216   Subject_1: 000232F-034-01   recKoRV:3

Query_1 320562
TTCCTATGTCCTCTCAGAAACTTTACACTCCAGTTGGGCTGATCTTCTCCCcatcCTCAGCACACAACTTATATGTGTATCTATATCCCCCCAA
CCCCTCAGGCC 320666

Subject_1 21036 ....
21090

Subject_1
27923 ....
............-....... 27975

Query_1: 000075F-038-01 Subject_1: phaCin_unsw_v4.1.fa.scaf00073 recKoRV:30

Query_1 10914 AAATATCCCAAATTTTCTCACAATATCTTATACCCACT-
GGGGTAGGTATActttAGGTTGGAAATCCTTGTTCTAAGTAATCTTCAGTCAGTACAGATATGAGC 11018

Subject_1 4193587 ....G................
4193642

Subject_1
4200471 ....
.... 4200524

Query_1: phaCin_unsw_v4.1.fa.scaf00237 Subject_1: 000255F-011-01 recKoRV:4

Query_1 3228356
TATAGTTCCATTTCCTAACAGACTAATTCAATGAGCTGGCTATCTAACTTTatggCATAATCTGAGCATAATGTATCTTATTCCACTTCTTTTT
CACATTGTGTA 3228460

Subject_1 5169 ....
5223

Subject_1
11094 ....
.... 11147

Query_1: phaCin_unsw_v4.1.fa.scaf00285 Subject_1: 000307F-027-01 recKoRV:5

Query_1 2864167
AACACGTATTTTAGAAATAAATTTATTAGGTGTCCATGTACCATCCAAGTActatCAGTATTTGTCTTCCATTTCTAAATACTATTGAAATGTG
GGGGGGGGCAG 2864271

Subject_1 644 ....
698

Subject_1
7520 ....
.... 7573

Query_1: scaffold592_size682540 Subject_1: phaCin_unsw_v4.1.fa.scaf00005 recKoRV:6

Query_1 429239
CCTGATTTGTTCTGGTTCCAGTACATGCAATCTCTCTTAGTTCACTGCTAacacACTTCCTAAACAATGGGGGTTCTGCCGACCTAGGGAGCCT
GTGAAA 429339

Subject_1 26679813 ....
26679759

Subject_1
26672907 ....
.... 26672858

Query_1: 000274F-009-01 Subject_1: phaCin_unsw_v4.1.fa.scaf00253 recKoRV:9

Query_1 591
TAGATACACTAACTAAATGTTTCAGAAATAAACAAAGGCTTTAAGTAAGTAgcagAACATTCAGCCCTGCCAAAATAATTAAAATGATAATACC
ATCAACATTAG 695

Subject_1 2925200 ....C.................................
2925254

Subject_1
2932089 ....
.... 2932142

Query_1: 000145F-047-01 Subject_1: phaCin_unsw_v4.1.fa.scaf00137 recKoRV2:1

Query_1 115
TTTTTGTTCATCTTTGCCAATTTGCATGGAGTGAAGTAAAACCTCAGAATTgtttTAATTTGCATTTTTCTATCTATTATCGATTTGGAGCAGC
ACCTTTCGCAG 219

Subject_1 4937987 ....
4938041

Subject_1
4945603 ....
.... 4945656

Query_1: phaCin_unsw_v4.1.fa.scaf00070 Subject_1: 000072F-131-01 recKoRV3:1

Query_1 2195367
CTGAGCTTTAGTTTTCACTTCTGCCACTTGGTTAAGAAAAATCTATTTCAGtaatTAATACAAGACAAAAGAACATGTGGTTAGATATCAGTCT
ACACATCAGTA 2195471

Subject_1 40854 ....
40908

Subject_1
47726 ....
.... 47779

B. ClustalW formatted multiple sequence alignment of integration sites from A above used as input to the sklign logo server, as described in the main text methods section. Each sequence in the alignment is restricted to a 24bp segment centred on the 4bp site which is duplicated in the integration.

CLUSTAL W (1.83) multiple sequence alignment

KoRV:10 TGAAGGGGTGctatCACTACAATT

KoRV:40 GGTCTTCCTGactcCAGAACCAGT

KoRV:9	GAGCTACATTgcgaAAAGCTCCAA
KoRV:12	TCAAAAGCTTatagAAGTGATTGC
KoRV:13	CTGGAGCATTacatGACTTGCTTT
KoRV:19	GTTGCAAGGTaaatGATTTTTGAG
KoRV:2	TAACTTTCAAgggtCAAAAATTAA
KoRV:21	TGTTTTGTAGgttcCTTCTTTGTA
KoRV:28	AGCCATGTTGtgcgATGACTAATG
KoRV:30	TTCCAGTCTGcttaCACCTTATTC
KoRV:39	GATTATATTGgtagATCATGCTAG
KoRV:46	CTTCAAGGTCctttCTGTGAAGAG
KoRV:5	TTGCCCCATTattgTATGGCTGAG
KoRV:16	TCTCTCACTCatagATTCCATAAC
KoRV:24	AAAAGTAATGctacAGGCATAACT
KoRV:43	AGGCCCCTGTgtacATGCTGAGAG
KoRV:48	AGCCAGGTAGgtagGTTGAGTAAG
KoRV:6	GGAAGGGTTCttagAAGCTATAGA
KoRV:7	GCATGTCACTgcctCAACCTTCTT
KoRV:8	CTTGGCTTTGgcagTAGGCTTAGA
KoRV:11	GGATAACATTatacTATTTACCTC
KoRV:14	GTCAGAAAACcagcAAAGTTTCCA
KoRV:15	AATGTTTATTgaacTAAGGGAAGG

KoRV:20	TATATATTGTgtatCTATATATTT
KoRV:22	GTGGTTTGTTgatgGATTCTTGTT
KoRV:25	GTTTAATATGctacCTATATTATA
KoRV:26	CATTTATTAAgtgcCTCTTATATG
KoRV:27	CATTAAGGACaaacAACAATAATG
KoRV:3	CATGTTAGTTgtatGACCCAGGGC
KoRV:31	TCAAAAAATCtttcATCCAACAGC
KoRV:33	GCCTGGGAAGtctcCTATGACCTG
KoRV:34	CACAAATATCatacAATATGACCA
KoRV:35	TCAGTTCTTTggatATCAGGTTCC
KoRV:42	GGAAGATTTTacagCATCTACAAA
KoRV:47	CAGTTTGGTTacagATGCTTAGAT
KoRV:51	TCCAGTCTTGaaatGAGCTAAGAG
KoRV:52	ATCTTTGGCCatggGTTTCTATAA
KoRV:53	TGGAGACTTTgtacAGTGGCAAGG
KoRV:56	GAGTTCCTTCatgtCTGCTGAAGT
KoRV:57	ATTTATTTTTccttGTCTTATAAT
recKoRV:10	CCTGGCAAAGggatCGTGTAGTCT
recKoRV:11	TGGAGTTCTTgtttCCAAAGCACA
recKoRV:2	TAAGAGCATCagatCAGATGATCT
recKoRV:3	ATCTTCTCCCcatcCTCAGCACAC

recKoRV:30	GGTAGGTATActttAGGTTGGAAA
recKoRV:4	ATCTAACTTTatggCATAATCTGA
recKoRV:5	CATCCAAGTActatCAGTATTTGT
recKoRV:6	TTCACTGCTAacacACTTCCTAAA
recKoRV:9	TAAGTAAGTAgcagAACATTCAGC
recKoRV2:1	CCTCAGAATTgtttTAATTTGCAT
recKoRV3:1	TCTATTTCAGtaatTAATACAAGA

Supplementary Figure 2

Endogenous KoRV-A entire genome, consensus tree, NJ method, no outgroup, 1000 Bootstrap showing recent radiation of KoRV-A in Koala Bilbo. (Blue = Koala Bilbo, Red= Reference KoRV-A Genomes).

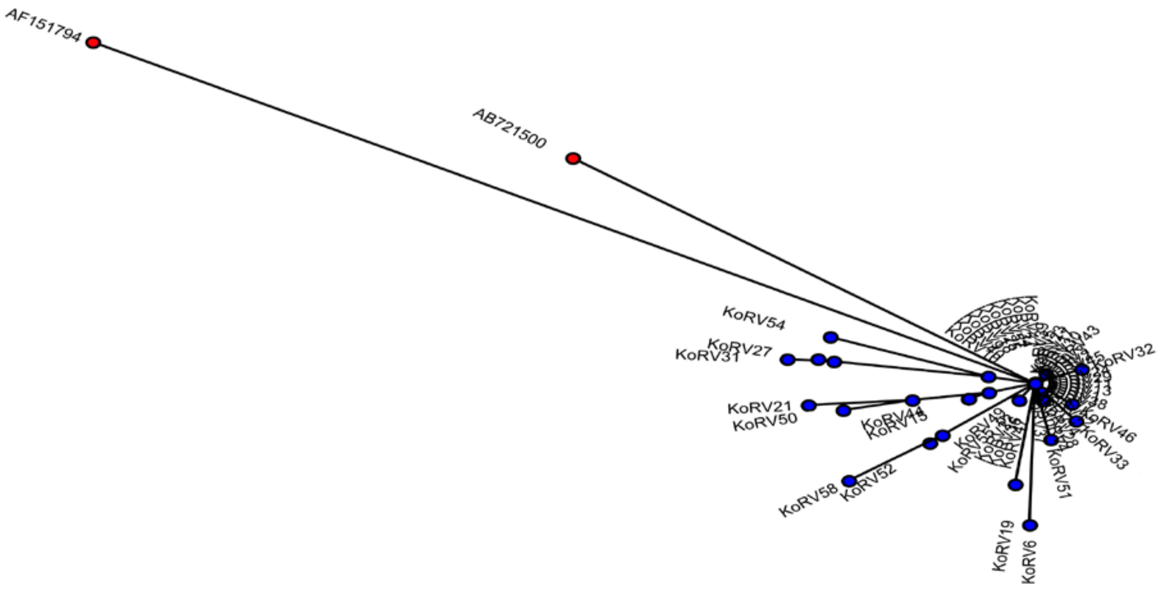

Supplementary Figure 3

PhER ("Phascolarctos endogenous retroelement")

A. Sequence listing (phaCin_unsw_v4.1.fa.scaf00062:10912078-10920108). Long terminal repeats (LTRs) are in upper case. A putative tRNA priming site is highlighted.

```
         10        20        30        40        50        60        70        80
----:----|----:----|----:----|----:----|----:----|----:----|----:----|----:----|
TGTAATGCAGAACATTATTTTACCATTTAGGTAGCAGAACCCTTTAGGCCATGGAAAACTCCAAGTTCCTTAGCAAGACC

         90       100       110       120       130       140       150       160
----:----|----:----|----:----|----:----|----:----|----:----|----:----|----:----|
CTTATCTGGCTGCTCAGGCTCCAGCCCTCAGATCATATCTCTGGAACCTCCAAATTCCTTAGCAAGACCCTTATCTGGCT

        170       180       190       200       210       220       230       240
----:----|----:----|----:----|----:----|----:----|----:----|----:----|----:----|
GCTCAGGCCACAGCCCTCAGATCATATCTCTGGAAGCCTCCATTGTGAGAAAGTTTGCAGAGCATTATCATATCTAGATA

        250       260       270       280       290       300       310       320
----:----|----:----|----:----|----:----|----:----|----:----|----:----|----:----|
GAGGGAGAGGGGTGTCCTTGACCAGCCCCCCCAAAAAAACTCTTCCTCTTGCCAGACTTCAAAGATCCATCCCCCTCCC

        330       340       350       360       370       380       390       400
----:----|----:----|----:----|----:----|----:----|----:----|----:----|----:----|
TGTCCTGTATACTCCCTATATAAACCCTGGCTCGACCCCTGATGAGGGCTGCTTGATTTGGATGTGAATATCCCTGGCAG

        410       420       430       440       450       460       470       480
----:----|----:----|----:----|----:----|----:----|----:----|----:----|----:----|
CCACGCGCTAATATAATAAAGTCCACAATTTAAATTAGTCTGGGTCAACTCGCTAATTTTTCGGTATAACAtttggagg

        490       500       510       520       530       540       550       560
----:----|----:----|----:----|----:----|----:----|----:----|----:----|----:----|
ccccagcgagatagcagcgtactgtgtcgctgcagagacccacctggagaggacatcggcctcgggaggggaattgttct

        570       580       590       600       610       620       630       640
----:----|----:----|----:----|----:----|----:----|----:----|----:----|----:----|
gttctgacttctaggggagccggcccctgatgatgttccagggccccaggactcaggactaggagaagcctccctccccg

        650       660       670       680       690       700       710       720
----:----|----:----|----:----|----:----|----:----|----:----|----:----|----:----|
actccgactgtacctcatttcggccctcgtggacctggtaaccctctggaggtaaatgtttgcaaacctgttttctggtg

        730       740       750       760       770       780       790       800
----:----|----:----|----:----|----:----|----:----|----:----|----:----|----:----|
gggaacgagtgccagacgtggcagagcacctaattagtgcctcaatcccccaggcaccccgagacgtcgggactatgcct

        810       820       830       840       850       860       870       880
----:----|----:----|----:----|----:----|----:----|----:----|----:----|----:----|
gtatctggttcctggtacccggtttctggttcctggtttctgtcttagttctgttgggcccgtgtctcgttcctggtttc

        890       900       910       920       930       940       950       960
----:----|----:----|----:----|----:----|----:----|----:----|----:----|----:----|
tgtctcagtgttggtctattagtttatatgtgtagagattgtgttttaagagattctgttttgcaatcatctgcctgaa

        970       980       990      1000      1010      1020      1030      1040
----:----|----:----|----:----|----:----|----:----|----:----|----:----|----:----|
gccacctctgtttcatactgaaagacaggtgtcagactggattgcagacagttttctccagggcagttcctttgacttca

       1050      1060      1070      1080      1090      1100      1110      1120
----:----|----:----|----:----|----:----|----:----|----:----|----:----|----:----|
ttgggaggatgtgtttgtgttcaggctcttattgtttgtctgtgtctgtgttctgcaataatgggggatacggccagta

       1130      1140      1150      1160      1170      1180      1190      1200
----:----|----:----|----:----|----:----|----:----|----:----|----:----|----:----|
catcatttccctccctttttgggttgtttgcagccagacagccaaaggctgcagcaggactctgaaaagttgccttcattc

       1210      1220      1230      1240      1250      1260      1270      1280
----:----|----:----|----:----|----:----|----:----|----:----|----:----|----:----|
tccccctctcctccccaggtaacagcagcttcaaagcagtgcgtggatgggagagggaaagagagaagaatactttatta

       1290      1300      1310      1320      1330      1340      1350      1360
----:----|----:----|----:----|----:----|----:----|----:----|----:----|----:----|
tttaggttatatggttggtagagggcaagttgggaaggattttgaagtgtcaggtcagaattagaaaggaatgagatat

       1370      1380      1390      1400      1410      1420      1430      1440
----:----|----:----|----:----|----:----|----:----|----:----|----:----|----:----|
gtacaggttttaaggggattttaaatcattagaaagaaaggcaaggaagtttaagcatgtaggagagaggagtgtgtgta

       1450      1460      1470      1480      1490      1500      1510      1520
```

```
----:----|----:----|----:----|----:----|----:----|----:----|----:----|----:----|
gaaaatttgaagggaatatgatttagttgcatggtgtgactggaggttttgaaagtgagaaggttttgggacttttgcat

       1530      1540      1550      1560      1570      1580      1590      1600
----:----|----:----|----:----|----:----|----:----|----:----|----:----|----:----|
gagtaatagtttctctctctctgcattctaactctggccaagtttgagtgtggagacaaagagaaaagttcttgtgtacc

       1610      1620      1630      1640      1650      1660      1670      1680
----:----|----:----|----:----|----:----|----:----|----:----|----:----|----:----|
agaacatagacaaatggtttatatgctgaggactacggcttgctgggtttagttgatatttcccacgagccccagctca

       1690      1700      1710      1720      1730      1740      1750      1760
----:----|----:----|----:----|----:----|----:----|----:----|----:----|----:----|
tccaaattggtaaatatgtgaggttttcaatttcagcatttgttacactgttttgcacttaggtacattgaaggaaactc

       1770      1780      1790      1800      1810      1820      1830      1840
----:----|----:----|----:----|----:----|----:----|----:----|----:----|----:----|
agacagtggattgttttgagtgcacagtgagggcagtttgaggagagatggagggaattatgtccacccacctttcaggc

       1850      1860      1870      1880      1890      1900      1910      1920
----:----|----:----|----:----|----:----|----:----|----:----|----:----|----:----|
tagatccccgggtgggttcttttaaagactggtcattatgggaaagaaaaagcaatcagaatactggtccagtacatttc

       1930      1940      1950      1960      1970      1980      1990      2000
----:----|----:----|----:----|----:----|----:----|----:----|----:----|----:----|
taagcttaacaagggtattgcatttgggaatatatttttgtctacccctccaaaaactaaataaataaataggggagaat

       2010      2020      2030      2040      2050      2060      2070      2080
----:----|----:----|----:----|----:----|----:----|----:----|----:----|----:----|
gaatgggttagaataaacttccaaattttattgcttttaagaggaagtttcacctataattcttatcaaatctgtgtaaa

       2090      2100      2110      2120      2130      2140      2150      2160
----:----|----:----|----:----|----:----|----:----|----:----|----:----|----:----|
cgttttctgtgttgtgaaattcctgagtaaatgagaattcttgagagataaaaattgagtaaattttaaaagtttgata

       2170      2180      2190      2200      2210      2220      2230      2240
----:----|----:----|----:----|----:----|----:----|----:----|----:----|----:----|
atatttgttattgtggtaatgttcttcacgcattataaaaactgaattttttctctgtttttgactaaggaaatttgat

       2250      2260      2270      2280      2290      2300      2310      2320
----:----|----:----|----:----|----:----|----:----|----:----|----:----|----:----|
gtctaggaattctgtaacaactaggaatttagtttgttatggtactttgggaagttatttatgttacttgaggacatttt

       2330      2340      2350      2360      2370      2380      2390      2400
----:----|----:----|----:----|----:----|----:----|----:----|----:----|----:----|
acatcaacttagttttaataattccattttaaaggtttatttttactttaaggtagaaaggaaagatatctatcacctt

       2410      2420      2430      2440      2450      2460      2470      2480
----:----|----:----|----:----|----:----|----:----|----:----|----:----|----:----|
aaaaatttctaacaaatgttcttcatgaaagtttagtgattattccttctaacatcaggataaactgtaagctttttag

       2490      2500      2510      2520      2530      2540      2550      2560
----:----|----:----|----:----|----:----|----:----|----:----|----:----|----:----|
aaagggaaatacttttttacaaagtaatgttttgcaaaatctttttgtatgatttttagaaggcctactaaatttccattt

       2570      2580      2590      2600      2610      2620      2630      2640
----:----|----:----|----:----|----:----|----:----|----:----|----:----|----:----|
gcaagctaatttgttgcaataatttaatactgataacaattatgtcttaatccactgtgaattttctaagtttcattcct

       2650      2660      2670      2680      2690      2700      2710      2720
----:----|----:----|----:----|----:----|----:----|----:----|----:----|----:----|
atgtaaatgtgtccttgaattggccttaaatgggtgctttcagagaaacagagtcaatggttcctgcttcaggtagcata

       2730      2740      2750      2760      2770      2780      2790      2800
----:----|----:----|----:----|----:----|----:----|----:----|----:----|----:----|
agaaagggcactggtctcaagaatgtaggtttgaaagttcaatgccccactctaagtttttaaggggtggacgtagaagt

       2810      2820      2830      2840      2850      2860      2870      2880
----:----|----:----|----:----|----:----|----:----|----:----|----:----|----:----|
gaaaaagataggacagctaaaacatcttagcagattctggaagtttttgagattgatcattaaggagtttttaaacacct

       2890      2900      2910      2920      2930      2940      2950      2960
----:----|----:----|----:----|----:----|----:----|----:----|----:----|----:----|
ccttacaatcttgtcactcctttcccccacttcctccccttgagtcaagagggtatctctgtgtggggatcccaaacagt

       2970      2980      2990      3000      3010      3020      3030      3040
----:----|----:----|----:----|----:----|----:----|----:----|----:----|----:----|
gctgggtttctctattaacccctttcagcccagatcagattaggaagagagtgttttacccccctctcccctgcccgcaa

       3050      3060      3070      3080      3090      3100      3110      3120
----:----|----:----|----:----|----:----|----:----|----:----|----:----|----:----|
attcaccttcccccacctggctataggaaggaaggggaggggcagcagctacagcctggaagtatcttccagcacacagc

       3130      3140      3150      3160      3170      3180      3190      3200
----:----|----:----|----:----|----:----|----:----|----:----|----:----|----:----|
catggggagtaaaaggggatgggtcaaggtaaagaagccagtctatagggatggagtggccccaggagactgttggacct

       3210      3220      3230      3240      3250      3260      3270      3280
```

```
----:----|----:----|----:----|----:----|----:----|----:----|----:----|----:----|
ggtgaaagggtctagaaagaagaaagatttttttattgggtcaattgtattagttatgttgctggaagggcaatttcttt

         3290      3300      3310      3320      3330      3340      3350      3360
----:----|----:----|----:----|----:----|----:----|----:----|----:----|----:----|
tataatctagaatgttaaattaattgtgtcttcaaaaaaaaaggagtcaatttggctgttggggcattttatatgatcct

         3370      3380      3390      3400      3410      3420      3430      3440
----:----|----:----|----:----|----:----|----:----|----:----|----:----|----:----|
gttttgtaacatttcctttaaatattgtaattcaaggaagtataaaatttcaaattttgtctctattgaaagggaatt

         3450      3460      3470      3480      3490      3500      3510      3520
----:----|----:----|----:----|----:----|----:----|----:----|----:----|----:----|
ttcagttgaaattgtttgttataatttataaaatttgtgagattgttaactaagttatttggggttattgttttaatatc

         3530      3540      3550      3560      3570      3580      3590      3600
----:----|----:----|----:----|----:----|----:----|----:----|----:----|----:----|
aaaacctaagattgttaattgattacttgtgtgtgaaaaggaggggcacatggcaattccccttattttgtaaacttgcc

         3610      3620      3630      3640      3650      3660      3670      3680
----:----|----:----|----:----|----:----|----:----|----:----|----:----|----:----|
aggatccctggaaactatggggggggggcaaggtcatctgctctgaaaaaaaaagtgcagcttccatatgtttgtttgac

         3690      3700      3710      3720      3730      3740      3750      3760
----:----|----:----|----:----|----:----|----:----|----:----|----:----|----:----|
atgtggcatgggaaggaatttcttaattgaatgggttgtaaattcaatagagcaggaaggtgtccaagtgtcagttgaa

         3770      3780      3790      3800      3810      3820      3830      3840
----:----|----:----|----:----|----:----|----:----|----:----|----:----|----:----|
attatagaccaagaaacatgacatagcatagtctatgtccatgagataccgaggtaagaaaagtcccattgttcgggaga

         3850      3860      3870      3880      3890      3900      3910      3920
----:----|----:----|----:----|----:----|----:----|----:----|----:----|----:----|
gtgtttggtcccagacatcttgggctcagtgataacaaacagaaagaatgttggtcagttctatctggtcctggaaaggg

         3930      3940      3950      3960      3970      3980      3990      4000
----:----|----:----|----:----|----:----|----:----|----:----|----:----|----:----|
gtgtttttggttcacttgaaaaaaaaaagaattagttcaggtttttggcccttattggggttcaattgattatactcacc

         4010      4020      4030      4040      4050      4060      4070      4080
----:----|----:----|----:----|----:----|----:----|----:----|----:----|----:----|
tcaagatgttcccattgtttaaaggaattatgagagcagtagtatttttctgtgatcagtctcttactattaccaatgtg

         4090      4100      4110      4120      4130      4140      4150      4160
----:----|----:----|----:----|----:----|----:----|----:----|----:----|----:----|
agattaaattcttattatctaggatttgtgattttatgggacagacatttactattgttttgagctttgattaccagggt

         4170      4180      4190      4200      4210      4220      4230      4240
----:----|----:----|----:----|----:----|----:----|----:----|----:----|----:----|
acttgtctgagttgtcataaagccaatagtagaatggcatgttctgcaggctgataactgataaaggtggaagtctgtgc

         4250      4260      4270      4280      4290      4300      4310      4320
----:----|----:----|----:----|----:----|----:----|----:----|----:----|----:----|
ctgggaagactttttgaggacaaccttggacacagcctagataggatcttcctgactctatttcccaattgtgctatttcc

         4330      4340      4350      4360      4370      4380      4390      4400
----:----|----:----|----:----|----:----|----:----|----:----|----:----|----:----|
tttctgacaagggaaatccccacctgattaatcccaggccctgcttttaacatccagtgactacgtgcttacctatcaga

         4410      4420      4430      4440      4450      4460      4470      4480
----:----|----:----|----:----|----:----|----:----|----:----|----:----|----:----|
tatgactcatgcctggaatcaaatggagctaaggaagttatatgatatatgaccctgtccctctggaacatccttgtgtt

         4490      4500      4510      4520      4530      4540      4550      4560
----:----|----:----|----:----|----:----|----:----|----:----|----:----|----:----|
accataataggatgcaagtacattcatggccccataataataatatagggataacatccccctaaggaggaaatgtttac

         4570      4580      4590      4600      4610      4620      4630      4640
----:----|----:----|----:----|----:----|----:----|----:----|----:----|----:----|
acacaataataggacaaaggtgtaatgtgatagatgtctaattaaataggatagcaaattttgagaaaagcaataggatt

         4650      4660      4670      4680      4690      4700      4710      4720
----:----|----:----|----:----|----:----|----:----|----:----|----:----|----:----|
agactgatatgattggcttccaagatgtatctatcatgcaatccaagctttaagcatgtttaggtaggaagatcacagtt

         4730      4740      4750      4760      4770      4780      4790      4800
----:----|----:----|----:----|----:----|----:----|----:----|----:----|----:----|
ttggatttttacactaagccagagaagcagagctccctttttgaactgtctatgaattaaacaaattgtacatgagcaag

         4810      4820      4830      4840      4850      4860      4870      4880
----:----|----:----|----:----|----:----|----:----|----:----|----:----|----:----|
gaacttaatgttagttaatataggtaatactgattgtattgatttgtatgattcctgtctaaggttttctttgttaccct

         4890      4900      4910      4920      4930      4940      4950      4960
----:----|----:----|----:----|----:----|----:----|----:----|----:----|----:----|
gattacatgtaaatctctcttttccaaactagtctattgtgagctctctgattggtttaatctgtacctgacttaatgta

         4970      4980      4990      5000      5010      5020      5030      5040
```

```
----:----|----:----|----:----|----:----|----:----|----:----|----:----|----:----|
attatgcaattttgcgaattatgagtaaactttgttgtattgtttgaacctagtcctgtgtgactgggaaaactgaacta

         5050      5060      5070      5080      5090      5100      5110      5120
----:----|----:----|----:----|----:----|----:----|----:----|----:----|----:----|
tctctatgtgcctttaactaaagacttatgagggattctggaatacttaggaattcagtttccttaatactactctggaa

         5130      5140      5150      5160      5170      5180      5190      5200
----:----|----:----|----:----|----:----|----:----|----:----|----:----|----:----|
tttgtgttatttaagaacttttgtaccaatttagtgaggttttatgaacaagagtagaaatgcatgcaagcattttaggg

         5210      5220      5230      5240      5250      5260      5270      5280
----:----|----:----|----:----|----:----|----:----|----:----|----:----|----:----|
ctcattttggaaatgttccttaggcaatgtgacatcttagggtactatagaactgggacttgtgttctgtgtgtgttcct

         5290      5300      5310      5320      5330      5340      5350      5360
----:----|----:----|----:----|----:----|----:----|----:----|----:----|----:----|
atatgagttaaggttctttacaattaggtcaggggtttgtgagaatgttattttgttatttgggtaatatcatacttgtt

         5370      5380      5390      5400      5410      5420      5430      5440
----:----|----:----|----:----|----:----|----:----|----:----|----:----|----:----|
taaagttgtgttacaattaataatgttaaaagggaaatggcttgtggtttatcttgtccatgtcatcagagaaacatggc

         5450      5460      5470      5480      5490      5500      5510      5520
----:----|----:----|----:----|----:----|----:----|----:----|----:----|----:----|
atgactgaaacatttaagatcctatatgcactgtgttaaaatggattctttcatatatctatgtatgtattggggcctgt

         5530      5540      5550      5560      5570      5580      5590      5600
----:----|----:----|----:----|----:----|----:----|----:----|----:----|----:----|
ggattaatgtaaggtatgcatattgttgtttgctcttgtttactatttagtggggggttggcagtatgggtccggctctt

         5610      5620      5630      5640      5650      5660      5670      5680
----:----|----:----|----:----|----:----|----:----|----:----|----:----|----:----|
acggcagtcctcttccttggatgattcctttgccaaaaactaaaagggggagtaggaactcagggcgatggcactaattc

         5690      5700      5710      5720      5730      5740      5750      5760
----:----|----:----|----:----|----:----|----:----|----:----|----:----|----:----|
ttccttaaccaagttcctgcagggactccagaaagaaaacacagaggccactactgatcatacttctcttgtgtttgttt

         5770      5780      5790      5800      5810      5820      5830      5840
----:----|----:----|----:----|----:----|----:----|----:----|----:----|----:----|
ggtccatgtttactaaatattcttgccagattcctttcttctagggtcggagccatcaacttccagatgactgtatacca

         5850      5860      5870      5880      5890      5900      5910      5920
----:----|----:----|----:----|----:----|----:----|----:----|----:----|----:----|
acccaaggtctctagtccactgaacctggcatcagccaggttttgaagtttgacccctcgaacgggacccagagaggcag

         5930      5940      5950      5960      5970      5980      5990      6000
----:----|----:----|----:----|----:----|----:----|----:----|----:----|----:----|
ggacagctctacgccccttgccagcaggaagcagtttcagaagcagagacctcccctttttcaccaaaagaatttggttcc

         6010      6020      6030      6040      6050      6060      6070      6080
----:----|----:----|----:----|----:----|----:----|----:----|----:----|----:----|
tatttgtttgagggggggatgatgggggcggggcccaaaccaagatcttcgctctttgcctcaaagggaatctgactcaca

         6090      6100      6110      6120      6130      6140      6150      6160
----:----|----:----|----:----|----:----|----:----|----:----|----:----|----:----|
ggccccagaccctcatctccctttgaaagccttagccttaccttttaatcttattcagatctggctccctcttgtctata

         6170      6180      6190      6200      6210      6220      6230      6240
----:----|----:----|----:----|----:----|----:----|----:----|----:----|----:----|
ggccccagaaaggccttcctttatctccttttgaaagtcctagccccaccttgattttgtgcagatgcctttcaaacttc

         6250      6260      6270      6280      6290      6300      6310      6320
----:----|----:----|----:----|----:----|----:----|----:----|----:----|----:----|
tgccttcctgttccctccattatctattcccattgtctattcaatctcccttgacccccctgcatttccctattctccct

         6330      6340      6350      6360      6370      6380      6390      6400
----:----|----:----|----:----|----:----|----:----|----:----|----:----|----:----|
acccctccctgtttcacatgcccccacctacttgcccatccccccctttcccttggttctccttccccttttcctctggctc

         6410      6420      6430      6440      6450      6460      6470      6480
----:----|----:----|----:----|----:----|----:----|----:----|----:----|----:----|
ctagtaggacccctcaggcagatcctcctcacccctcgattatgagactgctccgagccatccatagcgttgtcaactcc

         6490      6500      6510      6520      6530      6540      6550      6560
----:----|----:----|----:----|----:----|----:----|----:----|----:----|----:----|
acccggcctgacctaggacctagctgctggctgtgtatggatgcacaacccccatactatgtgggagtggcagttaatgg

         6570      6580      6590      6600      6610      6620      6630      6640
----:----|----:----|----:----|----:----|----:----|----:----|----:----|----:----|
ttctgtttcccatagcccagactcggaaaattgtcggtgggaacaacctaagttgaccctgggggatgtccaaggaaaag

         6650      6660      6670      6680      6690      6700      6710      6720
----:----|----:----|----:----|----:----|----:----|----:----|----:----|----:----|
gtacctgtcttacctcatccaccacatccctgaaaacttctccttactctcctgtctgtaatagtactgtggttgttccc

         6730      6740      6750      6760      6770      6780      6790      6800
```

```
----:----|----:----|----:----|----:----|----:----|----:----|----:----|----:----|
caatcctcctcctcttttattcagcaccatccggcacttggtgggcctgtctggatgagatcacacagtgtgtctccgc

         6810      6820      6830      6840      6850      6860      6870      6880
----:----|----:----|----:----|----:----|----:----|----:----|----:----|----:----|
tgtggtgttcctaaaagaatctgagcctctatgtatattagtctccattatccccagggcccggagggttgggagtactt

         6890      6900      6910      6920      6930      6940      6950      6960
----:----|----:----|----:----|----:----|----:----|----:----|----:----|----:----|
cactcatgagggcagtgtatcctactgaagcaagcgagccgttcccctcctcatccctatcctcagtggggttggggtta

         6970      6980      6990      7000      7010      7020      7030      7040
----:----|----:----|----:----|----:----|----:----|----:----|----:----|----:----|
gtgggatcaactgctgtgggtaccgctgccctagtccgaggagaggccagttaccaggaacttagtgctcaggtcaacgt

         7050      7060      7070      7080      7090      7100      7110      7120
----:----|----:----|----:----|----:----|----:----|----:----|----:----|----:----|
agacctcagtcatctggagcactctatatcccaactggagagacaggtcaactccttggcagagatggttctgcagaacc

         7130      7140      7150      7160      7170      7180      7190      7200
----:----|----:----|----:----|----:----|----:----|----:----|----:----|----:----|
ggaggggtttggacttactgtttctcaggcaaggtggcctttgtgccgccctaggggaggcctgctgtttctatgccaat

         7210      7220      7230      7240      7250      7260      7270      7280
----:----|----:----|----:----|----:----|----:----|----:----|----:----|----:----|
aactcagacgtagttcaggagtacctttccctagtacggaaaaatattgcagacaggcagagggaacgagaagggaacga

         7290      7300      7310      7320      7330      7340      7350      7360
----:----|----:----|----:----|----:----|----:----|----:----|----:----|----:----|
aaattggtaccagagcctgtttcggacttccccatggttaaccattcttatttctgccttagccgggcctttgctcctcc

         7370      7380      7390      7400      7410      7420      7430      7440
----:----|----:----|----:----|----:----|----:----|----:----|----:----|----:----|
ttactatagccctaattcttgacccttgtctagttaatcggctccttgactttgttagatcctgtattaatttagttaag

         7450      7460      7470      7480      7490      7500      7510      7520
----:----|----:----|----:----|----:----|----:----|----:----|----:----|----:----|
ttactgttggttagaggtccccgttatcagaccctggcgtctaccttctttgcaccctataatgaggcagcccccaccaa

         7530      7540      7550      7560      7570      7580      7590      7600
----:----|----:----|----:----|----:----|----:----|----:----|----:----|----:----|
gtcaagggtttggtggtacacaaaagaagtggggaaTGTAATGCAGAACATTATTTTACCATTTAGGTAGTAGAACCCTT

         7610      7620      7630      7640      7650      7660      7670      7680
----:----|----:----|----:----|----:----|----:----|----:----|----:----|----:----|
TAGGCCATGGAAAACTCCAAATTCCTTAGCAAGACCCTTATCTGGCTGCTCAGGCTCCAGCCCTCAGATCATATCTCTGG

         7690      7700      7710      7720      7730      7740      7750      7760
----:----|----:----|----:----|----:----|----:----|----:----|----:----|----:----|
AAACCTCCAAATTCCTTAGCAAGACCCTTATCTGGCTGCTCAGGCCACAGCCCTCAGATCATATCTCTGGAAGCCTCCAT

         7770      7780      7790      7800      7810      7820      7830      7840
----:----|----:----|----:----|----:----|----:----|----:----|----:----|----:----|
TGTGAGAAAGTTTGCAGAGCATTATCATATCTAGATAGAGGGAGAGGGGTGTCCTTGACCAGCCCCCCCAAAAAAAAAA

         7850      7860      7870      7880      7890      7900      7910      7920
----:----|----:----|----:----|----:----|----:----|----:----|----:----|----:----|
CTCTTCCTCTTGCCAGACTTCAAAGATCCATCCCCCTCCCTGTCCTGTATACTCCCTATATAAACCCTGGCTCGACCCCT

         7930      7940      7950      7960      7970      7980      7990      8000
----:----|----:----|----:----|----:----|----:----|----:----|----:----|----:----|
GATGAGGGCTGCTTGATTTGGATGTGAATATCCCTGGCAGCCACGCGCTAATATAATAAAGTCCACAATTTAAATTAGTC

         8010      8020      8030
----:----|----:----|----:----|-
TGGGTCAACTCGCTAATTTTTCGGTATAACA
```

B. blastx alignments

Query ID

lcl|Query_242870

Description

PhERV phaCin_unsw_v4.1.fa.scaf00062:10912078-10920108

Query Length

8031

Database Name

nr

Description

All non-redundant GenBank CDS translations+PDB+SwissProt+PIR+PRF excluding environmental samples from WGS projects

Three alignments:

```
>XP_014403529.1 PREDICTED: endogenous retrovirus group S71 member 1 Env polyprotein-like,
partial [Myotis brandtii]
Length=447

 Score = 240 bits (613),  Expect = 2e-65, Method: Compositional matrix adjust.
 Identities = 136/298 (46%), Positives = 201/298 (67%), Gaps = 4/298 (1%)
 Frame = +1

Query  6412  PQADPPHPSIMRLLRAIHSVVNSTRPDLGPSCWLCMDAQPPYYVGVAVNGSVSHSPDSEN  6591
              P +     S++ +L  +H  +N+++P +G  CWLC+D +PPYYVGV ++  +     + N
Sbjct  150   PHSPSQESSLLEMLGTVHQFINTSQPTVGRDCWLCLDPRPPYYVGVGISQDIPQFQGTSN  209

Query  6592  CRWEQPKLTLGDVQgkgtcltssttslktspyspVCNSTVVVPQssssfysapsGTWWAC  6771
              C W+QP+LTLGD+QG+G+CL S    L+ SPY+  CN T     S +++Y AP  TW AC
Sbjct  210   CSWDQPRLTLGDLQGQGSCLLSPNFPLQRSPYADSCNQTHWGNSSFTTYYQAPEQTWLAC  269

Query  6772  LDEITQCVSAVVFLKESEPL-CILVSIIPRARRVGSTSLMRAVYPTEASEPF---Psssl  6939
               + +T+C S+ +F +  +PL C+LV I+P+            +Y   +S  +   P
Sbjct  270   TNGLTKCASSDLFKEGEDPLLCVLVYILPQIFIYNGNEGRAHMYSGLSSNRYKRAPVLVP  329

Query  6940  ssvglglvgsTAVGTAALVRGEASYQELSAQVNVDLSHLEHSISQLERQVNSLAEMVLQN  7119
                + LG+ GSTA+G  AL++G+   +ELS QV++D+ HLE SIS LE+QV+SLAE+VLQN
Sbjct  330   LLISLGIAGSTAIGALALIKGDMDLRELSKQVDMDIRHLESSISHLEKQVDSLAEVVLQN  389

Query  7120  RRGLDLLFLRQGGLCAALGEACCFYANNSDVVQEYLSLVRKNIADRQREREGNENWYQ  7293
              RRGLDLLF++QGGLC ALGE CCFYANNS +++E L+LVR+N+ +R+R+RE +E W+Q
Sbjct  390   RRGLDLLFMKQGGLCMALGETCCFYANNSGIIRESLALVRRNLEERERKREQSETWFQ  447


>CAI15393.1 T envelope protein [Pongo pygmaeus]
Length=625

 Score = 233 bits (595),  Expect = 3e-61, Method: Compositional matrix adjust.
 Identities = 158/347 (46%), Positives = 216/347 (62%), Gaps = 11/347 (3%)
 Frame = +1

Query  6430  HPSIMRLLRAIHSVVNSTRPDLGPSCWLCMDAQPPYYVGVAVNGSVSHSPDSENCRWEQP  6609
               PS+M +L  +H ++N T+P L   CWLC+ A+PPYYVG+ V  ++   P S  C
Sbjct  276   QPSLMSILAGVHHLLNLTQPKLAQDCWLCLKAKPPYYVGLGVEATLKRGPLS--CYTRPR  333

Query  6610  KLTLGDVQgkgtcltssttslktspyspVCNSTVVVPQssssfysapsGTWWACLDEITQ  6789
               LTLGDV G  +CLTS+  +L  SP+   CN +++   S+S  Y AP  TW AC   +T+
Sbjct  334   ALTLGDVSGNASCLTSTGYNLSASPFQATCNQSLLTSISTSVSYQAPDNTWLACTSGLTR  393

Query  6790  CVSAVVFLKESEPL-CILVSIIPRARRVGSTSLMRAVYPTEAS----EPFPssslssvgl  6954
              C++      E  PL C+LV ++P+            + P E         P        L
Sbjct  394   CINGT----EPGPLLCVLVHVLPQVYVYSGPEAQLLIAPPELHPRLHRAVPLLVPLLADL  449

Query  6955  glvgsTAVGTAALVRGEASYQELSAQVNVDLSHLEHSISQLERQVNSLAEMVLQNRRGLD  7134
               + GS A+G AALV+GE     LS QV+VDLS+L+ +I  L  QV SLAE+VLQNR+GLD
Sbjct  450   SIAGSAAIGMAALVQGETGLMSLSQQVDVDLSNLQSAIDILHSQVESLAEVVLQNRQGLD  509

Query  7135  LLFLRQGGLCAALGEACCFYANNSDVVQEYLSLVRKNIADRQREREGNENWYQSLFRTSP  7314
              LLFL QGGLCAALGE+CCFYAN S V+++    VR+N+  RQ+ERE N  WYQS+F  +P
Sbjct  510   LLFLSQGGLCAALGESCCFYANQSGVIEDTFQKVRENLDRRQQERENNIPWYQSMFNWNP  569

Query  7315  WLTilisalagpllllltialilDPCLVNRLLDFVRSCINLVKLLLVR  7455
              WLT LI+ LAGPLL+L ++LIL PC++N  L+F++  I  VKL+ ++
Sbjct  570   WLTTLITGLAGPLLILLLSLILGPCILNWFLNFIKQRIASVKLMYLK  616


>XP_015421279.1 PREDICTED: retrovirus-related Env polyprotein from Fv-4 locus
[Myotis davidii]
Length=474

 Score = 221 bits (563),  Expect = 1e-58, Method: Compositional matrix adjust.
 Identities = 131/358 (37%), Positives = 214/358 (60%), Gaps = 20/358 (6%)
 Frame = +1

Query  6424  PPHPS---------IMRLLRAIHSVVNSTRPDLGPSCWLCMDAQPPYYVGVAVNGSVSHS  6576
              PP PS         + + +   + ++N++RP+L  SC LC+DA+PPYY G  + G  + +
```

```
Sbjct  105   PPTPSHSLPSNADLLWKTVEGAYRILNASRPELTKSCCLCLDAKPPYYEGFTIKGDYTAA  164

Query  6577  PDSENCRWEQ--PKLTLGDVQgkgtcltssttslktspyspVCNSTVVVPQssssfysap  6750
               + +CRW+Q   +LTL  V         +      S  S +CN T+  P++++
Sbjct  165   STTSSCRWQQTAARLTLQAV-----TGQGTCIGNVPSEQSHLCNETLATPKAAAYLLPPE  219

Query  6751  sGTWWACLDEITQCVSAVVFLKESEPLCILVSIIPR-ARRVGSTSLMR--AVYPTEASEP  6921
                WWAC    +  C+ ++V   + E  CILV +IP+     G   L R  A+      EP
Sbjct  220   -NAWWACSSGLAPCIHSLVLNSQKEGFCILVQLIPKLIYHSGEEVLNRIDAINSRSKREP  278

Query  6922  FPssslssvglglvgsTAVGTAALVRGEASYQELSAQVNVDLSHLEHSISQLERQVNSLA  7101
               + +++++    +     G ++LV   + +Y  L A +++D+  +E SI+ L+  + SL+
Sbjct  279   ITALTVAALLGLGLVGARTGISSLVIQDKNYGALRAAIDLDIERIEKSITHLQESLTSLS  338

Query  7102  EMVLQNRRGLDLLFLRQGGLCAALGEACCFYANNSDVVQEYLSLVRKNIADRQREREGNE  7281
              E+VLQNRRGLDLLF++QGGLCAALGE CCFY ++S VV+E ++LVR+ +  R+ ERE ++
Sbjct  339   EIVLQNRRGLDLLFMQQGGLCAALGEECCFYVDHSGVVKETMALVREGLQKRKLEREQSQ  398

Query  7282  NWYQSLFRTSPWLTilisalagpllLltialilDPCLVNRLLDFVRSCINLVKLLLVR  7455
              +WY+ LF  SPWLT L+SAL GPL++L + L   PC++N+L+ FV+  I +V+L++++
Sbjct  399   SWYEFLFNWSPWLTTLVSALVGPLVILLMLLTFGPCIINKLVQFVKEHIGIVQLMVLQ  456
```

Supplementary Figure 4. PCR primers used in KoRV-B, KoRV-D and KoRV-E specific PCR

Primer	Seq 5'- 3'	Tm° C	length	position	target
**UKoRV-F**	AGTCCTGGGAACTGGAAAAGAC	59	22	5948-5969 AF151794	KoRV Env - Universal
**UKoRV2-R**	TGCCAGGCTCGGTGAACTC	61	19	6542-6524 AF151794	KoRV Env - Universal
**KoRVBK1-R**	TTGCCTGACTAACCCCCTGC	58	20	6335-6315 KC779547 (modified)	KoRV-B RBD - Universal
**KoRVBK1-F**	GCCAGAATCTCAACAGTCTGC	58	21	6257-6277 KC779547	KoRV-B RBD - Universal
**KoRVE Bilbo 2F**	CTTTGCATAACGGGCCTTGG	58	20	Bilbo specific primers	KoRV-E RDB - Bilbo specific
**KoRVE Bilbo 2R**	CCCGTTGAACTGTAAAGCCAGG	58	22	Bilbo specific primers	KoRV-E RDB - Bilbo specific
**KoRVD Bilbo 3F**	CCAAGCCACCTTTGTTTGACC	58	21	Bilbo specific primers	KoRV-D RDB - Bilbo specific
